# Tunable metafibers: remote spatial focus control using 3D nanoprinted holograms on dual-core fibers

**DOI:** 10.1038/s41377-025-01903-0

**Published:** 2025-07-07

**Authors:** Jun Sun, Wenqin Huang, Adrian Lorenz, Matthias Zeisberger, Markus A. Schmidt

**Affiliations:** 1 Department of Fiber Photonics, Leibniz Institute of Photonic Technology, 07745 Jena, Germany; 2 Abbe Center of Photonics and Faculty of Physics, Friedrich-Schiller-University Jena, 07743 Jena, Germany; 3 Otto Schott Institute of Materials Research (OSIM), Friedrich-Schiller-University Jena, 07743 Jena, Germany

**Keywords:** Nanophotonics and plasmonics, Fibre optics and optical communications, Sub-wavelength optics, Integrated optics

## Abstract

The generation of tunably focused light at remote locations is a critical photonic functionality for a wide range of applications. Here, we present a novel concept in the emerging field of *Metafibers* that achieves, for the first time, fast, alignment-free, fiber-integrated spatial focus control in a monolithic arrangement. This is enabled by 3D nanoprinted intensity-sensitive phase-only on-fiber holograms, which establish a direct correlation between the intensity distribution in the hologram plane and the focus position. Precise adjustment to the relative power between the modes of a dual-core fiber generates a power-controlled interference pattern within the hologram, enabling controlled and dynamic focus shifts. This study addresses all relevant aspects, including computational optimization, advanced 3D nanoprinting, and tailored fiber fabrication. Experimental results supported by simulations validate the feasibility and efficiency of this monolithic *Metafiber* platform, which enables fast focus modulation and has transformative potential in optical manipulation, high-speed laser micromachining, telecommunications, and minimally invasive surgery.

## Introduction

The focusing of light is a fundamental photonic functionality, enabling the precise redirection, strong spatial concentration, and localized detection of optical power for efficiently exploiting light-matter interaction, which is essential across various fields. It serves as a prerequisite for many optical applications such as telecommunications (e.g., wavelength division multiplexing^1^), medicine (e.g., laser ablation^2^), biophotonics (e.g., optogenetics^3,4^), and materials science (e.g., laser micromachining^5^).

Here, dielectric nanostructures represent a topical research direction for light focusing^6^, with two primary strategies currently being pursued: Metasurfaces (MSs) consist of arrays of subwavelength elements with tailored phase and amplitude properties that allow for complex beam manipulation, e.g. for the generation of orbital angular momentum beams^7^. The second strategy is phase-only holograms, which offer more efficient beam manipulation through reduced structural complexity, resulting in better mechanical stability, lower fabrication costs, and higher diffraction efficiency. Note that such holograms allow highly efficient control of the phase and intensity distribution of the focused light, whereas polarization shaping requires metasurfaces which use anisotropic subwavelength elements with controlled amplitude and phase of the orthogonal polarization eigenstates. As a result, phase-only holograms are generally preferred when polarization shaping is not required, having found applications in fields such as optical tweezers^8^, augmented reality^9^, and metrology^10^.

Many of the mentioned applications require flexible guidance and focusing of light at remote locations, which is generally difficult to achieve with bulk optics. Step-index optical fibers provide an excellent solution for efficient and flexible light transport, having revolutionised photonics in various fields such as telecommunications^11^, quantum technologies^12^, and sensing^13^.

The interfacing of planar nanophotonic structures with optical fibers defines the research field of *Metafibers*, enabling precise beam shaping and light manipulation directly at the fiber output. Unlike metasurfaces, which are typically fabricated on planar substrates, *Metafibers* integrate functional photonic nanostructures onto the fiber itself for enhanced light control. A key factor in the advancement of *Metafiber* research has been the advent of 3D nanoprinting, which, unlike conventional lithography, is compatible with the fiber geometry and introduces a new level of freedom by individually controlling the height of each element^14,15^. Noteworthy examples of *Metafiber* research include phase-only holograms for optical tweezers^16^, phase-controlled multifocal arrays^17^, MS-based structures for achromatic light focusing^18^ and complex beam shaping^19^, as well as structures for boosting fiber coupling at large angles^20–22^.

All mentioned fiber devices ultimately produce a static focus at a fixed focal position, which is disadvantageous for applications requiring real-time variable focus manipulation^23^, such as optical traps and optical tweezers^24^, multiplexing in telecommunications^25^, nanoscale bioanalysis^26^ and laser-based 3D nanoprinting and micromachining^5,27^.

Approaches for real-time modulation of optical fiber output are generally divided into two categories: External modulation approaches, such as spatial light modulators^28–30^, adaptive optics^31^, mechanical microactuators^32^, and digital micromirrors^33^, provide ways to adjust the output light, but are slow and not fiber-integrated or fiber-interfaced, limiting efficiency and application range. Internal modulation approaches based on thermo-optical tuning (e.g., liquid core fibers^34^) or magneto-optical effects (e.g., ferrofluids^35^ or liquid crystals^36^) allow very limited modulation of the output beam and are also slow. These limitations significantly underscore the urgent need for advanced modulation technologies and innovative approaches in fiber optics.

Multicore fibers with single-mode cores and suppressed modal crosstalk represent a key area of research in fiber optics today, as they address technological challenges such as increasing telecommunication data rates^37^, scaling fiber laser power^38,39^, imaging complex tissues^40^, and creating novel high-efficiency sensors^41,42^. As a result, the combination of fibers that include multiple cores with phase-only holograms is expected to open up new applications, particularly for tunable remote light focusing.

**Fig. 1:**
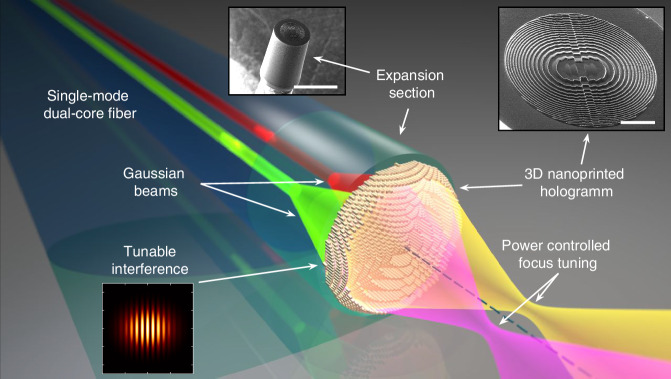
Illustration of the Tunable Metafiber concept, enabling power-controlled, fully fiber-integrated spatial focusing using a phase-only 3D nanoprinted hologram coupled to a single-mode dual-core fiber. To illustrate the functional principle, two focusing light beams related to two relative power differences of the guided modes (green and red) are shown by the yellow and red magenta areas (dashed dark blue line: central fiber axis). The lower left inset shows an example of the intensity distribution of the interfered Gaussian beams in the hologram plane when the power in the modes is equal. The top right (scale bar: 20 µm) and center (scale bar: 100 µm) insets show images of the nanoprinted 3D hologram and the expansion section on the end face of the dual-core fiber, respectively

In this work, we present a novel approach that demonstrates, for the first time, power-controlled, a tunable *Metafiber* allowing for fully fiber-integrated spatial focus control using a phase-only hologram coupled to a single-mode dual-core fiber (Fig. 1). Key to the approach is a 3D nanoprinted intensity-profile sensitive hologram that establishes a relationship between the intensity distribution in the hologram plane and the focus position in the image plane. By precisely tuning the relative modal amplitudes (i.e., respective guided powers) of the fundamental modes of the fiber cores, a power-controlled interference pattern is generated within the hologram plane, resulting in a spatial shift of the focus. This study encompasses the entire design process, including computational optimization, device fabrication using 3D nanoprinting, and fiber fabrication. The experimental validation, supported by simulations, demonstrates the feasibility and effectiveness of this monolithic all-fiber concept.

## Results

**Fig. 2:**
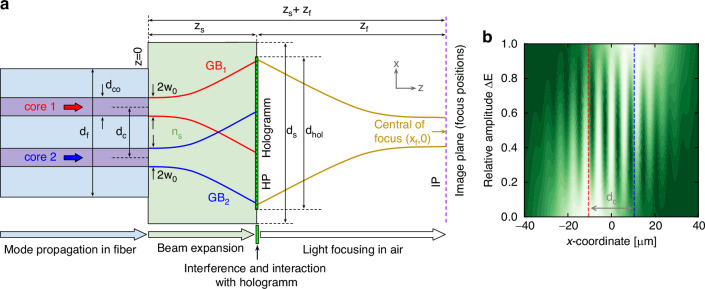
Cross-section of the fully fiber-integrated device for spatial focus control and associated intensity distribution. **a** Visualization of the cross-section of the all-fiber focus tuning concept (xz-plane) including the relevant parameters. The horizontal arrows at the bottom of the sketch indicate the light-matter interaction in the respective section of the device (light blue: mode propagation in the dual-core fiber, light green: beam expansion in the homogeneous spacer, green: interference of the two GBs and interaction with the hologram, white: light focusing in air). The red and blue lines illustrate the two Gaussian beams (GB_1_: red, GB_2_: blue) interfering in the hologram plane (HP, green, $z={z}_{{\rm{s}}}$). The vertical dashed violet lines refer to the image plane (IP) where the light (dark yellow lines) is focused ($z={z}_{{\rm{s}}}+{z}_{{\rm{f}}}$). **b** Intensity distribution along the x-axis within the HP at y = 0 for various relative amplitude differences Δ*E* (linear scale from 0 (dark green) to unity (white)). Each distribution is normalized to its maximum value. The vertical dashed red and blue lines indicate the central positions of the core, i.e. GBs. The simulation parameters correspond to those of the experiment [see Supplementary Note 12, table. S3]

The presented concept (Fig. 2a) is based on a step-index dual-core fiber (DCF) interfaced with a nanoprinted phase-only hologram, with the cores sufficiently spaced to avoid modal crosstalk (${d}_{{\rm{c}}}=21\,{\rm{\mu m}}$, Fig. 2a, section “mode propagation”). One side of this fiber is connected to a spacer of homogeneous material used for beam expansion (Fig. 2a, section “expansion”). The top of the spacer hosts the phase hologram (hologram plane (HP), $z={z}_{{\rm{s}}}$), which allows to focus the light in the image plane (IP, $z={z}_{{\rm{s}}}+{z}_{{\rm{f}}}$) (Fig. 2a, section “focusing”).

The functional principle of the tuning concept discussed here is based on (i) the interference of expanded Gaussian beams (GBs) resulting from the fundamental modes of the fiber cores in the HP and (ii) the phase-only hologram that is sensitive to the input intensity profile. It is important to note that for a constant phase difference between the modes, the interference pattern of the superimposed GBs (Fig. 2b) depends mainly on the relative amplitude difference between the two modes. In this context the parameters ${E}_{1}$ and ${E}_{2}$ represent the amplitudes of the modes in core 1 and core 2 respectively and are defined with respect to the relative amplitude difference $\varDelta E$ as ${E}_{1}=\varDelta E$ and ${E}_{2}=1-\varDelta E$. The parameter Δ*E* controls the relative contribution of the mode and lies in the range $0\leqslant \varDelta E\leqslant 1$. At $\varDelta E=0$, all power is confined to mode 2, while the complementary situation is achieved for $\varDelta E=1$. For intermediate values, the beams of the modes interfere in teh HP to produce a distinct intensity distribution, which is governed by the period of the spatial intensity oscillation ${X}_{{\rm{p}}}$ [details in Supplementary Note 1]. Note that to achieve sufficient overlap of the electric fields of the two GBs, the distance between HP and fiber surface was chosen to be significantly larger than the Rayleigh length (${z}_{R}=21\,{\rm{\mu m}}$, ${z}_{s}=290\,{\rm{\mu m}}$, $\frac{{z}_{{\rm{s}}}}{{z}_{{\rm{R}}}}\approx 13.6$).

The DCFs used were fabricated in-house and consist of two GeO_2_-doped cores in a fused silica matrix (refractive index contrast 6 × 10^−3^, all relevant parameters in this study are listed in Table S3). The cores show a higher-order mode cut-off at approximately 584 nm and microbend-induced losses starting at 1100 nm (c.f. Methods Section and Supplementary Note 4, Fig. S5), resulting in a single-mode operation interval of $600\,{\rm{nm}} \, < \, {\lambda }_{0}\, < \, 1000 \, {\rm{nm}}$. Modal crosstalk at the operating wavelength (${\lambda }_{0}=660\,{\rm{nm}}$) was effectively suppressed by careful choice of the core parameters and the intercore distance (${d}_{{\rm{c}}}=21\,{\rm{\mu m}}$, centre-to-centre). The numerical aperture and mode field radius (i.e., beam waist) of both cores were determined by characterizing the output radiation (${w}_{0}=1.5 \, {\rm{\mu m}}$, NA = 0.139, [c.f. see Supplementary Note 3, Fig. S4], showing identical properties of both cores.

**Fig. 3:**
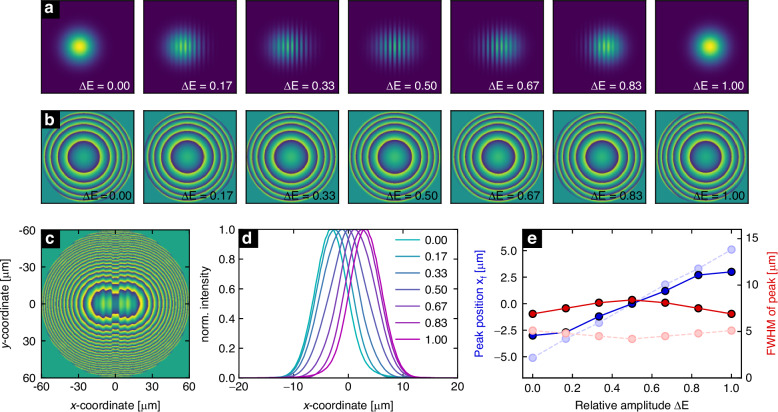
Design steps of the phase-only hologram and associated focus properties. Number of holograms considered here is ${N}_{f}=7$. **a** Calculated intensity distributions of the interfered GBs (linear scale, dark: 0, bright: 1, each plot is normalized to its maximum value) in the HP for various relative amplitude differences $\varDelta E$ (indicated in the lower right corners). Each plot has a size of 120 µm × 120 µm. **b** Associated phase distributions (120 µm × 120 µm, linearly scaled from $-\pi ...+\pi$) obtained using the ASM-based GSA^78^. **c** Combined phase distribution resulting from the superposition of the fields of the individual holograms and the phase correction (same scale as (b)). **d** Intensity distribution in the IP along the x-axis (${y}_{{\rm{f}}}=0$) for the different value of relative amplitude differences (each colour refers to one value of $\varDelta E$, c.f. legend). **e** Peak position (blue dots, left y-axis) and FWHM (red dots, right y-axis) of each focus as a function of $\varDelta E$ (obtained from direct analysis of the data points). The semi-transparent blue and red dots refer to the corresponding values resulting from the individual holograms (c.f. (b)). The solid and dashed lines are only included to connect the dots and act as visual guides

The design strategy to obtain the intensity-profile sensitive phase-only hologram in the HP essentially relies on the superposition of the phase distributions of the individual holograms obtained for different combinations of input intensity distribution (i.e., different relative amplitude differences $\varDelta E$) and focus positions in the IP. In detail, the strategy consists of the following steps (simulation parameters in Table S3), which represent the complete design framework and ensure precise reproducibility of the procedure:

Step 1: (Fig. 3a): Calculation of the intensity distribution in the HP for different input intensity distributions of interfered GBs ${I}_{i}^{{\rm{GB}}}$ (Fig. 2a, $i=1,...,{N}_{f}$), i.e. relative amplitude differences $\varDelta E$.

Step 2: Calculation of intensity profiles in the IP (focus coordinates ${r}_{i}^{{\rm{f}}}=({x}_{i}^{{\rm{f}}},{y}^{{\rm{f}}}=0)$). Note that the assumed foci are defined by 2D Gaussian distributions with very small radii (${w}_{{\rm{f}}}=1 \, {\rm{\mu }}m$).

Step 3: (Fig. 3b): Determination of the phase distribution of each hologram ${\phi }_{i}({x}_{i},y=0)$ for the different combinations of $\varDelta E$ and ${r}_{i}^{{\rm{f}}}$ by iterative phase retrieval. Specifically, the Gerchberg-Saxton algorithm (GSA) has been applied using the Angular-Spectrum-Method (ASM) for forward and backward propagation, resulting in a general approach that is not limited to Fraunhofer or Fresnel domains. The spatial resolution was set to $\varDelta x,\varDelta y=300 \, {\rm{nm}}$, which corresponds to the voxel size in 3D nanoprinting. A sufficiently large simulation domain was chosen to avoid edge effects. The resulting phase distributions (Fig. 3(b)) show kinoform distributions shifted along the x-direction, which is ultimately due to the very small diameter of the foci assumed in the IP (${w}_{{\rm{f}}}=1\,{\rm{\mu }}m$), resulting in a highly symmetric superimposed hologram, which is advantageous for 3D nanoprinting.

Step 4: The phase distribution of the final hologram ${\phi }_{{\rm{hol}}}$ is determined by the superposition of the electric fields of all configurations in the HP. Each field is defined by ${E}_{i}^{{\rm{HP}}}=\sqrt{{I}_{i}^{{\rm{HP}}}}\cdot \exp \left(i\cdot {\phi }_{i}\right)$, leading to the total field ${E}_{{\rm{tot}}}^{{\rm{HP}}}$ given by:

1 $${E}_{{\rm{tot}}}^{{\rm{HP}}}=\mathop{\sum }\limits_{1}^{{N}_{F}}{E}_{i}^{{\rm{HP}}}={E}_{{\rm{hol}}}\cdot \exp \left(i\cdot {\phi }_{{\rm{hol}}}\right)$$

with the amplitude ${E}_{{\rm{hol}}}$ and the phase hologram ${\phi }_{{\rm{hol}}}$. Note that the superposition of electric fields is used here instead of the phases, which follows the standard procedure in holography and is also used in other holography-related contexts^43^.

Step 5: (Fig. 3c): In the final step, the intrinsic curvature of the wavefronts of the GBs within the HP is compensated for. This is achieved by superimposing the fields of all interfered GBs at $z={z}_{{\rm{s}}}$,

2 $${E}_{{\rm{tot}}}^{{\rm{GB}}}=\mathop{\sum }\limits_{1}^{{N}_{{\rm{hol}}}}{E}_{i}^{{\rm{GB}}}\left(z={z}_{{\rm{s}}}\right)={E}_{{\rm{tot}},0}^{{\rm{GB}}}\cdot \exp \left(i\cdot {\phi }_{{\rm{tot}}}^{{\rm{GB}}}\right)$$

with the total amplitude and phase ${E}_{{\rm{tot}},0}^{{\rm{GB}}}$ and ${\phi }_{{\rm{tot}}}^{{\rm{GB}}}$. The latter is subtracted from the previously determined phase distribution, resulting in the final phase hologram (Fig. 3(c):

3 $$\phi ={\phi }_{{\rm{hol}}}-{\phi }_{{\rm{tot}}}^{{\rm{GB}}}$$

This hologram is compensated for the average phase of all GBs in the HP and contains the phase contributions of all individual holograms, leading to a highly symmetric phase distribution.

An extensive sweep was performed to determine the optimal parameters of the hologram. Specifically, to determine configurations that result in optimal performance, the parameter sweep aims to find configurations that balance maximizing spatial modulation, minimizing full width at half maximum (FWHM) of the focal spot, ensuring identical focal spots for all relative amplitude differences Δ*E*, suppressing side peak amplitudes, and maximizing spatial separation of the side peaks from the central focal spot (details can be found in Supplementary Note 7).

To demonstrate the focusing properties of the combined phase hologram, the foci in the IP were calculated from the different input intensity distributions ${I}^{{\rm{GB}}}\left(\varDelta E\right)$ using the ASM in forward mode (Fig. 3d). Each input intensity distribution results in a focus in the IP that is shifted along the x-axis. To quantify the tuning characteristics, the peak position ${x}_{{\rm{f}}}$ and the full width at half maximum (FWHM) of each focus were determined (Fig. 3e), showing that ${x}_{{\rm{f}}}$ strongly shifts with $\varDelta E$ (blue dots). The maximum achievable shift is about 5 µm for the combined hologram, which is below the predefined shift of 10 µm (semi transparent blue dots). This effect results from the superposition of the individual phase distributions, which affects the function of each individual hologram. The associated FWHM remains nearly constant for all values of $\varDelta E$ and is slightly higher for the combined phase hologram (mean of all values 7.6 μm) than for the individual holograms (mean 4.7 μm).

To implement the hologram experimentally, the determined phase distribution needs to be transferred to a nanopatterned height-modulated surface consisting of nanoprinted dielectric elements with sub-wavelength dimensions that precisely manipulate the phase of passing waves. The height of these elements is calculated using^16^:

4 $$h\left(x,y\right)=\phi \left(x,y\right){\cdot \lambda }_{0}/\left(2\pi \left({n}_{{\rm{p}}}-1\right)\right)$$

with refractive index of the polymer ${n}_{{\rm{p}}}$.

The *Metafibers* were fabricated by 3D nanoprinting the designed holograms on the cleaved end face of the DCFs (see Methods section) using a process optimized for high spatial resolution, structural strength, and mechanical coupling to the DCF. The spacer section had a diameter larger than the DCF to avoid light reflections and a length (290 µm) longer than the Rayleigh length to ensure efficient interference of the GBs in the HP (details in Supplementary Note 12). The optical setup includes coupling optics, the *Metafiber*, and diagnostics. Monochromatic light is split into two beams to excite the modes in the DCF, and the resulting focus profile is measured with a camera (see Methods section). The characterization process involves adjusting the relative power difference between the beams and capturing the corresponding beam profile in the IP. Data analysis involved fitting the measured intensity distribution to an Airy function (see Supplementary Note 10 for derivation of the Airy function) to determine key focus parameters, including focus position and transverse extent in the xy-plane (see Methods section for details).

**Fig. 4:**
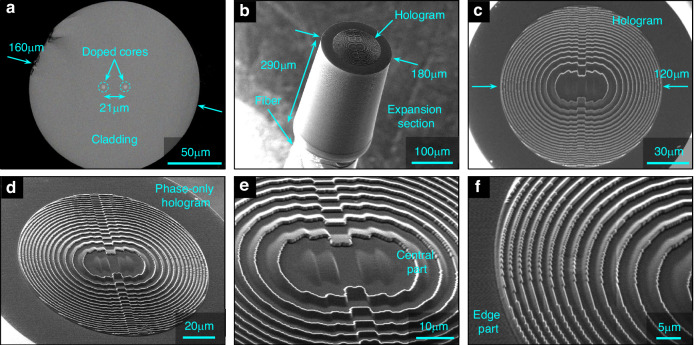
SEM images of the implemented phase-only holograms on the end face of a DCF. **a** Bare end face of the DCF. **b** Oblique view of the entire fiber tip including nanoprinted spacer and hologram. **c** Corresponding top view. **d** Tilted view showing the entire hologram. **e** Close-up of the central part of the hologram. **f** Enlarged view of the edge area of the hologram

A selection of SEM images of the implemented device, i.e., the nanoprinted hologram on the end face of the DCF are shown in Fig. 4 (fabrication details can be found in the Method Section). The exceptionally high quality of the hologram is clearly visible (Fig. 4c, d), both in the central area (Fig. 4e) as well as in the edge domains (Fig. 4f): no irregularities can be observed, while the structure accurately resembles the simulated phase distribution $\phi (x,y)$ (i.e., the calculated height profile $h(x,y)$. In addition, the hologram is precisely aligned with the center of the spacer (Fig. 4c). Another notable feature is the larger diameter of the spacer compared to the outer diameter of the fiber (Fig. 4a, b), as well as the conical support at the bottom of the spacer, which overlaps the fiber and strengthens the mechanical connection (c.f. inset of Fig. 6).

**Fig. 5:**
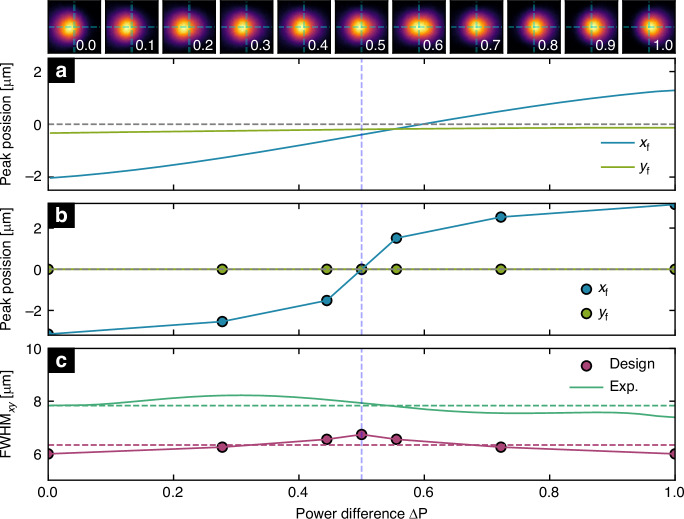
Experimental results of fully fiber-integrated power-controlled spatial focus control. The focus tuning characteristics were obtained by fitting the measured intensity distribution by an Airy function. **a** Measured peak position in the xy-plane (blue: x-direction, green: y-direction) as a function of the relative power difference $\varDelta P$. The top row shows selected examples of measured intensity distributions in the vicinity of the focus for the values of $\varDelta P$ (indicated in the lower left corner). **b** Plot showing the corresponding values obtained from the design and simulations. In both plots, the horizontal dashed grey lines refer to the centre lines at x = 0 or y = 0. **c** Comparison of the evolution of the mean width of the peaks ${{\rm{FWHM}}}_{{\rm{xy}}}$ as a function of $\varDelta P$ measured in the experiments (light green) and resulting from the design (red). The dashed lines show the corresponding mean values (${{\rm{FWHM}}}_{\exp ,{\rm{mean}}}=7.8\,{\mu m}$, ${{\rm{FWHM}}}_{{\rm{design}},{\rm{mean}}}=6.7\,{\mu m}$). Note that the curves are obtained by fitting an Airy function to the intensity distributions, while the results shown in Fig. 3e are obtained by direct analysis of the data points along the x-direction. In all plots, the vertical dashed light blue lines refer to $\varDelta P=0.5$. A detailed study of the wavelength dependence, which includes the influence of wavelength and material dispersion on the Gaussian beam interference and intensity distribution in the focal plane, confirms that the tuning of a high-quality focus is maintained over a spectral bandwidth of 100 nm (details in Supplementary Note 9)

The experimental demonstration of the focus tuning properties is based on exciting the fundamental modes of the two cores with defined relative power differences and measuring the resulting intensity distribution at the IP (Fig. 5a). A significant focus shift is observed along the x-axis, with a minimal shift along the y-axis, while each value of $\varDelta P$ leads to an almost Gaussian intensity distribution (top row in Fig. 5). This behaviour closely matches the simulated results (Fig. 5b), while the slight variation along the y-axis is likely due to small misalignments between the fiber axis, spacer, and hologram in the HP. Note that the curves in Fig. 5 correspond to the relative power difference, $\varDelta P$, rather than the relative amplitude difference, $\varDelta E$, which leads to the slight S-shape of both experimental and simulated curves (c.f. Supplementary).

The focal spot size was determined for the different intensity distributions using the fitting method described above and shows an almost constant focus width across different configurations (green curves in Fig. 5c). This result is in good agreement with the theoretically calculated curves (red curves in Fig. 5c). The mean of all measured focus width values is ${{\rm{FWHM}}}_{{\rm{xy}},{\rm{mean}}}^{\exp }=7.8\,{\rm{\mu m}}$ (green dashed horizontal line in Fig. 5C), which is about 1 µm higher than the simulated value (${{\rm{FWHM}}}_{{\rm{xy}},{\rm{mean}}}^{{\rm{design}}}=6.3\,{\rm{\mu m}}$, red dashed horizontal line in Fig. 5c). The measured focal length is ${z}_{{\rm{f}},\exp }=510\,{\rm{\mu m}}$, which compares well with the design focal length of ${z}_{{\rm{f}},{\rm{design}}}=520\,{\rm{\mu m}}$. The resulting geometric numerical apertures can be calculated via ${\rm{NA}}=\sin (\arctan (\frac{{d}_{{\rm{hol}}}}{2{z}_{{\rm{f}}}}))$ with ${d}_{{\rm{hol}}}=120\,{\rm{\mu m}}$, yielding ${{\rm{NA}}}_{\exp }=0.117$ and ${{\rm{NA}}}_{{\rm{design}}}=0.115$, which are very close, confirming the high quality of the implemented structure. The small deviation between the experimental and simulated data can be attributed to differences between the designed and actual experimental structures, as well as to a slightly suboptimal phase correction (${\phi }_{{\rm{tot}}}^{{\rm{GB}}}$). Overall, the experiments clearly validate the proposed concept and design methodology.

## Discussion

The tunable *Metafiber* concept presented is based on an intensity-profile sensitive phase hologram created by 3D nanoprinting on the end surface of a DCF. Further research could concentrate on increasing the intensity sensitivity of the hologram, possibly using advanced optimization algorithms, such as novel phase retrieval algorithms with improved properties (e.g., weighted Yang–Gu algorithm^44^, gradient descent methods^45^, or deep-learning approaches^44^ or inverse design strategies that take into account the peculiarities of 3D nanoprinting^46^. In addition to intensity modulation, future research will address other modulation parameters such as phase or polarization distribution within the HP. Exploring the tuning of more complex intensity distributions in the IP, such as arrays of foci^17^ or images, represents another promising area for future research and could pave the way for new applications. More advanced photonic functionalities such as longitudinal focus tuning or numerical aperture control are future research directions. While there are no fundamental physical limitations, the key challenge is to determine whether intensity profile sensitive tuning can effectively enable these capabilities.

DCFs play a central role in the discussed concept, as they generate the intensity-sensitive interference patterns in the HP. Increasing the flexibility of this concept can be achieved by exploring DCF with different parameters (e.g., higher core doping concentration, smaller core diameters). The design presented in this work has been optimized to ensure high quality foci for all relative power differences. Note that additional simulations indicate that a larger spatial focus tuning range can be achieved at the expense of focus quality (see Supplementary Note 8 for an example), requiring future studies to fully explore this trade-off. The capabilities of the concept can be further enhanced by increasing the number of cores, allowing the creation of more complex intensity patterns^47^. Recent advances in fiber fabrication have shown significant reductions in modal crosstalk at higher core densities, achieved by embedding individual cores in materials with suppressed refractive indices^48^.

The concept presented is based on power modulation, which is inherently faster than modulation methods using mechanical manipulation^49^, liquid elements^50^, liquid crystal metastructures^51^ or adaptive-optical element systems^52^. It should be noted that the potential modulation speed of the approach presented here is in the range of recently reported electro-optical devices (Fig. 1(a) in ref. ^23^.) and thus faster than most other approaches. Fast power modulation can be principally achieved by adjusting the power of either foci at the input of the DCF using techniques such as electro-optic^53^ or acousto-optic modulation^54^, or direct laser current modulation^55^. In addition, spatial modulation of the input beam, for example using a spatial light modulator^56^, can also provide a suitable approach to power modulation. Compared to our previous study on hologram enhanced multicore fibers^57^, this work introduces intensity profile sensitive holograms for continuous focus tuning instead of creating discrete focal spots. The DCF approach also enables ultrafast modulation and seamless fiber network integration, unlike our previous study, which relied on a fixed core-focus relationship in a 37-core fiber.

The discussed concept for remote focus manipulation using a computationally optimized hologram that is interfaced with SM-MCF is temperature stable over a realistic temperature range (details can be found in Supplementary Note 11) and has significant potential for various applications. The relevant scenarios can be divided into forward (light emission) and backward operation (light collection). In forward mode, the device principally provides fast modulation of optical trapping potentials (e.g., dynamic control of micro- and nanoparticles^58^ or time-averaged potential^59^ or control of resonant excitation (e.g., resonances in micro- and nanophotonic systems^60,61^. In addition, the tunable focus shift can allow for selective mode excitation in multimode waveguide systems, supporting transitions between fundamental and higher order modes, which is critical in telecommunications^62^ and nonlinear photonics^63^. In backward operation, the device is relevant for spatially resolved detection of nano-object motion (e.g., tracking of single rapidly diffusing objects in nanoparticle tracking analysis in life science^64^ and characterization of nanomaterials^65^ or supporting high-throughput cell flow analysis (e.g., enabling rapid 1D spatially resolved detection of fluorescence^66^. From the general perspective, focus manipulation enables precise and rapid particle control without the need for mechanical components, making it ideal for biological and microscopic applications^67^. In addition, in high-speed laser micromachining, the ability to quickly adjust the focus can improve the precision and throughput of manufacturing processes^68^. The system could enhance optical signal processing in telecommunications by improving mode control in fiber networks^69^ or in-coupling in integrated photonics^70^. Moreover, precise and fast focus modulation can be applied to laser surgery, offering increased accuracy and safety^71^.

Compared to established light field manipulation using high-NA multimode fibers^24^, our approach offers several differences, including a unique operating principle that eliminates the need for complex and costly coupling devices while enabling inherently fast operation by avoiding electronic latency. In addition, our single-mode design is independent of intermodal coupling, ensuring temperature insensitivity and eliminating the need for calibration. The use of silica-based materials further enhances mechanical and thermal robustness while maintaining compatibility with existing fiber circuits, enabling seamless integration through established methods such as photonic lanterns and mode-selective couplers^72^, waveguide-based fan-in/fan-out devices^73^, tapered and fusion joint single-mode fibers^74^, and lens system-based coupling^74^.

The tunable generation of focused light at remote locations is a critical photonic functionality that is fundamental to a wide range of optical applications. In this work, we have presented a novel approach that demonstrates, for the first time, fast power-controlled, fully fiber-integrated spatial focus control using a phase-only hologram interfaced to a single-mode DCF. Central to this approach is a 3D nanoprinted intensity-profile sensitive hologram that establishes a direct correlation between the intensity distribution in the hologram plane and the focus position in the image plane. By adjusting the relative power between the fundamental modes of the two fiber cores, a power-controlled interference pattern is generated in the hologram plane, resulting in a controlled spatial shift of the focus. Our comprehensive study covers the entire design process using computational optimization for holographic phase hologram retrieval. The implementation of this monolithic all-fiber device was achieved through advanced 3D nanoprinting techniques and complex fiber fabrication. Experimental verification, along with detailed optical characterization, demonstrated a total focus shift of more than 3 µm while maintaining focus shape, all being in close agreement with simulations.

In summary, this work presents for the first time a Tunable *Metafiber* consisting of a novel, monolithic, all-fiber device that enables rapid modulation of focus characteristics solely through power control, potentially surpassing the performance of conventional devices. This innovative concept holds great promise for applications in fields such as optical manipulation for biology and microscopy, nanoscale material analysis, high-speed laser micromachining, nanophotonics, telecommunications, integrated photonics and laser surgery.

## Methods

### Fiber implementation

The dual-core fibers were fabricated using the stack-and-draw technique, which included doped and undoped tube fabrication, hexagonal stacking, and fiber drawing (details can be found in Supplementary Information of ref. ^75^). The final fiber has an outer diameter of ${d}_{{\rm{f}}}=125\,{\rm{\mu m}}$ and a center-to-center intercore distance of ${d}_{{\rm{c}}}$ = 21 µm.

### 3D nanoprinting

The structures were fabricated using a commercial 3D nanoprinter (Photonic Professional GT2, Nanoscribe GmbH, Germany) by two-photon absorption polymerization of IP-Dip2 photoresist. The direct laser writing system used a 63× objective (NA = 1.4) and achieved spatial resolutions of $\varDelta x,\varDelta y=300{\rm{nm}}$ laterally and 1000 nm longitudinally (details available in refs. ^16,76^). During development, unpolymerized resin was removed by immersing the fiber end in propylene glycol monomethyl ether acetate (PGMEA) for 30 min, followed by a 12 min immersion in isopropanol under UV illumination. This process not only eliminated the PGMEA, but also enhanced polymerization, improving the strength of the structures.

### Optical setup and measurement procedure

The optical setup for characterising the focus-tuning concept consists of three main parts: coupled optics, sample with hologram, and diagnostics for analysing the intensity distribution at the IP (Fig. 5). Monochromatic light from a laser (660 nm laser diode, Thorlabs LP660-SF20) is split into two power- and polarization-controlled beams, which are used to excite the fundamental mode in one of the cores of the dual-core fiber. After passing through the sample and hologram, the intensity distribution and output power are measured using a camera and a power meter (PM). The optical characterization is based on adjusting the relative power difference $\varDelta P$ in the arms before the sample and measuring the intensity distributions in the IP by taking camera images. The procedure includes the following steps: (i) placing the PM at position 1 and coupling the two beams split at BS1 into the two fiber cores by adjusting the optics in front of the sample (i.e. objective OBJ1, beam splitters BS1 and BS2, mirrors M1 and M2), (ii) finding the image plane in the camera by adjusting OBJ2, and (iii) placing the power meter at position 2 and rotating the half-wave plates HW3 and HW4 to obtain the maximum power of both beams. This procedure makes it possible to achieve the same polarization of the beams inside the fiber cores.

**Fig. 6:**
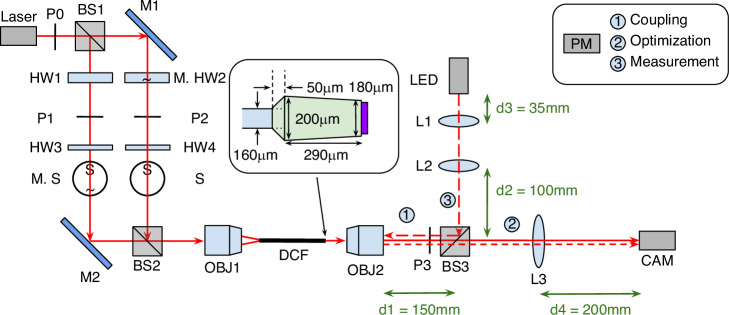
The optical setup for fully fiber-integrated spatial focus control. The setup was used to characterize the power-controlled focus tuning concept (LASER: laser diode (Thorlabs LP660-SF20), P: linear polarizer (Thorlabs LPVISC050-MP2), BS: beam splitter (Thorlabs BS013), (M.)HW: (motorized) half-waveplate (Thorlabs AHWP05M-600), M.S: motorized beam shutter (Thorlabs SH05R/M), S: beam shutter, M: mirror (Thorlabs PF10-03-P01), OBJ1: objective (Olympus PLN40X), OBJ2: objective (Olympus UPlanFLN 20X), L1: lens (Thorlabs LB1811-B, bi-convex f = 35 mm), L2: lens (Thorlabs LA1461-B, plano-convex f = 250 mm), L3: lens (Thorlabs LB1945-B, bi-convex f = 200 mm), CAM: camera (IDS UI-3252LE-C), PM: powermeter (Thorlabs PM100A with S120C sensor). The inset in the middle shows the end of the DCF including the colonial spacer (light green) and the hologram (purple). The inset in the upper right corner refers to the three different positions of the power meter (PM) according to the respective measurement performed (c.f. numbers in the setup)

For the actual measurement, the PM is placed in position 3 to monitor the transmitted power during image acquisition. The curve sign marked on the components (M. HW2 and M.S) in Fig. 6 means they are electrically motorised and programmed. The measurement procedure consists the following steps: (i) measuring the power range of the right beam by rotating the motorised half waveplate, (ii) measuring the power of the left beam and finding the to-be-measured angles (from the angle-power curve of the motorised half waveplate) that give the power ratio difference of 1%, (iii) sweeping the motorised half waveplate through the found angles, at which the power of the right beam is confirmed again (by closing the motorised beam shutter and measuring power) and then the focus profile is captured with both beams switched on. Note that the single motorised beam shutter and half waveplate setup is a cost efficient approach, which can be easily updated by placing a pair of motorised beam shutter and half waveplate along each beam.

### Data analysis

To determine the key focus parameters (i.e., focus position ${r}_{{\rm{f}}}=({x}_{{\rm{f}}},{y}_{{\rm{f}}})$ and transverse extent), the measured intensity distribution in the IP and the corresponding simulation data were fitted to an Airy function^16,76^, which resembles the shape of an ideal focus. In detail, the intensity distribution is modelled as:

5 $$I\left(x,y\right) \sim {{\rm{jinc}}}^{2}\left[0.9806\pi \sqrt{{\left(\frac{x-{x}_{{\rm{f}}}}{{{\rm{FWHM}}}_{{\rm{x}}}}\right)}^{2}+{\left(\frac{y-{y}_{{\rm{f}}}}{{{\rm{FWHM}}}_{{\rm{y}}}}\right)}^{2}}\right]+{A}_{{\rm{c}}}$$

where ${{\rm{FWHM}}}_{{\rm{x}}}$ and ${{\rm{FWHM}}}_{{\rm{y}}}$ represent the FWHM extent of the focus in the x- and y-directions, respectively, leading to the overall FWHM in the xy-plane: ${\rm{FWHM}}=\left({{\rm{FWHM}}}_{{\rm{x}}}+{{\rm{FWHM}}}_{{\rm{y}}}\right)/2$. Note that the parameter ${A}_{{\rm{c}}}$ refers to an intrinsic noise offset that must be taken into account when fitting the experimental data.

## Supplementary information

Supplementary Information for ''Tunable Metafibers: Remote Spatial Focus Control Using 3D Nanoprinted Holograms on Dual-Core Fibers''

## Data Availability

The data and code (DOI^77^) supporting this study can be downloaded from Zenodo.
