# Supplementary material for: Tunable metafibers: remote spatial focus control using 3D nanoprinted holograms on dual-core fibers
Source: Light Sci Appl. 2025 Jul 7;14:237. doi: 10.1038/s41377-025-01903-0 (PMC12234834; doi:10.1038/s41377-025-01903-0)
Supplement: Supplementary file 1 — Supplementary Information for ''Tunable Metafibers: Remote Spatial Focus Control Using 3D Nanoprinted Holograms on Dual-Core Fibers'' [file 41377_2025_1903_MOESM1_ESM.pdf]

# Supplementary Information for *"Tunable Metafibers: Remote Spatial Focus Control Using 3D Nanoprinted Holograms on Dual-Core Fibers"*

*Authors*

Jun Sun[1], Wenqin Huang[1], Adrian Lorenz[1], Matthias Zeisberger[1], Markus A. Schmidt[123]*

*Affiliations*

[1]Department of Fiber Photonics, Leibniz Institute of Photonic Technology, 07745 Jena, Germany.

[2]Abbe Center of Photonics and Faculty of Physics, Friedrich-Schiller-University Jena, 07743 Jena, Germany.

[3]Otto Schott Institute of Materials Research (OSIM), Friedrich-Schiller-University Jena, 07743 Jena, Germany.

*Corresponding author. Email: markus-alexander.schmidt@uni-jena.de

## Supplementary Note 1. Interference of two Gaussian beams

*A. Definition of Gaussian beam*

In a medium of refractive index $n_s$, the electric field $E(x,y,z)$ of a Gaussian beam with a waist $w_0$ is given by:

$$E(x,y,z) = E_0 \frac{w_0}{w(z)} \cdot \exp\left[-\frac{x^2+y^2}{w(z)^2}\right] \cdot \exp\left[-ikz - ik\frac{x^2+y^2}{2R(z)} + i\zeta(z)\right] \qquad \text{(Eq. S1)}$$

where $k = 2\pi n_s/\lambda_0$ is the wave number in the medium of refractive index $n_s$, and $\zeta(z) = \arctan(z/z_R)$ is the Gouy phase shift with the Rayleigh range $z_R$ given by

$$z_R = \frac{\pi \cdot w_0^2 n_s}{\lambda_0} \qquad \text{(Eq. S2)}$$

The radius of the wave front curvature and the radius of the beam waist of the beam at location z are described by

$$R(z) = z\left(1 + \left(\frac{z_R}{z}\right)^2\right) \qquad \text{(Eq. S3)}$$

$$w(z) = w_0\sqrt{1 + \left(\frac{z}{z_R}\right)^2}$$

These equations collectively describe the evolution of a Gaussian beam as it propagates through the medium.

*B. Approximations in the context of this work*

In the present work, the relevant distances at which interference occurs (in the HP) are significant larger than the Rayleigh length of the beam ($z_R = 16.58\ \mu m$, $z = 290\ \mu m$, $z_R << z_s$, vertical gray dashed line in Fig. S1), allowing to approximate the above-mentioned parameters of the Gaussian beam as follows:

$$R(z) = z\left(1 + \left(\frac{z_R}{z}\right)^2\right) \approx z \qquad \text{(Eq. S4)}$$

$$w(z) = w_0\sqrt{1 + \left(\frac{z}{z_R}\right)^2} \approx \frac{w_0}{z_R} z$$

As can be seen from Fig. S1, these equations approximate the situation addressed in this study to a very high degree.

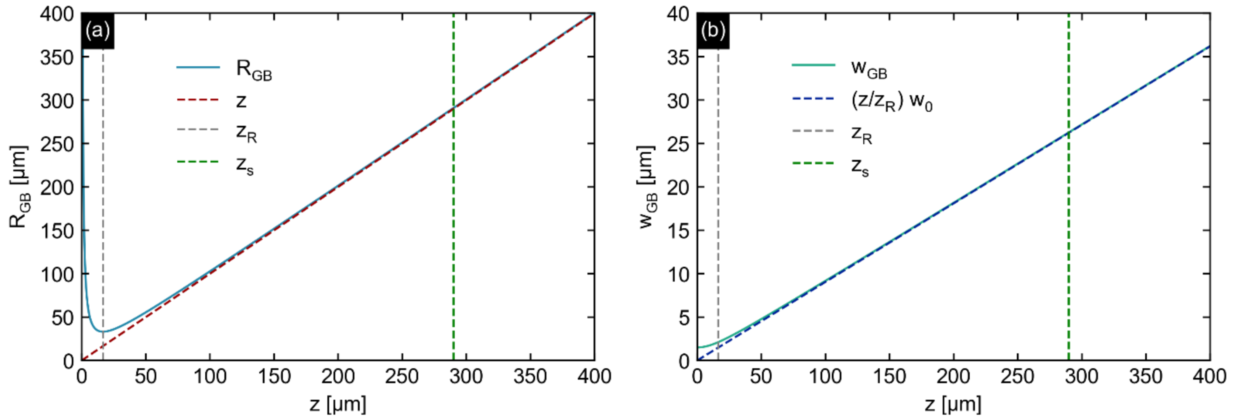


**Figure S1: Dependence of the main parameters of a Gaussian beam as a function of the propagation distance and related approximations.** Wavefront curvature and beam radius evolve as a function of the distance from the beam waist (located at $z = 0\ \mu\text{m}$) and their corresponding approximations (dashed lines) used in the context of the present study. (**a**) wavefront curvature. (**b**) beam radius. The curves were calculated for $w_0 = 1.5\ \mu\text{m}$, $\lambda_0 = 0.66\ \mu\text{m}$, $n_s = 1.548$, resulting in a Rayleigh length of $z_R = 16.58\ \mu\text{m}$ (vertical grey lines). The vertical dashed green line refers to the length of the spacer ($z_s = 290\ \mu\text{m}$) used in this work.

*C. Derivation of analytic equation for intensity distribution of two interfering co-propagating Gaussian beams*

To derive an analytical equation for the intensity distribution of two co-propagating Gaussian beams ($I_{GB}$) that interfere at location $z$, we first consider the total electric field and then calculate the intensity using $I_{GB} = E_{tot} * E_{tot}^*$. The total electric field can be expressed as the superposition of the electric fields of the individual Gaussian beams at location $z$:

$$E_{tot} = \Delta E \cdot E_1 + (1 - \Delta E) \cdot E_2$$

$$= Q_1 \cdot Q_3 \cdot [-\exp(Q_2 \cdot (d_c + 2x)^2) \cdot (\Delta E - 1) + \exp(Q_2 \cdot (d_c - 2x)^2) \cdot \Delta E] \quad \text{(Eq. S5)}$$

where $Q_1 = \frac{w_0}{w}$, $Q_2 = \frac{ik}{8R} - \frac{1}{4w^2}$, $Q_3 = \exp\,[i(kz - \phi_G)]$, and $\Delta E$ is the relative amplitude difference. When calculating the total intensity, it is important to note that the phase factor

disappears since $Q_3 \cdot Q_3^* = 1$. The second factor in front of the parentheses is entirely real-valued ($\mathrm{Im}(Q_1) = 0$), leading to $Q_1 \cdot Q_1^* = \left(\frac{w_0}{w}\right)^2$. Thus, the remaining expression for the intensity depends only on the term in parentheses, leading to the definition:

$$f = -\exp[Q_2 \cdot (d_\mathrm{c} + 2x)^2] \cdot (\mathit{\Delta}E - 1) + \exp[Q_2 \cdot (d_\mathrm{c} - 2x)^2] \cdot \mathit{\Delta}E \qquad \text{(Eq. S6)}$$

The intensity of the interfered beams can now be expressed as:

$$I_\mathrm{GB} = Q_1^2 \cdot (f \cdot f^*) \qquad \text{(Eq. S7)}$$

The parameter $Q_2$ is a complex value whose conjugate is given by:

$$Q_2^* = -\frac{ik}{8R} - \frac{1}{4w^2} \qquad \text{(Eq. S8)}$$

which leads to:

$$Q_2 + Q_2^* = -\frac{1}{2w^2} \qquad \text{(Eq. S9)}$$

The function $f$ consists of two parts that can be conjugated separately:

$$\begin{gathered} \{-\exp[Q_2 \cdot (d_\mathrm{c} + 2x)^2] \cdot (\mathit{\Delta}E - 1)\}^* = -\exp[Q_2^* \cdot (d_\mathrm{c} + 2x)^2] \cdot (\mathit{\Delta}E - 1) \\ \{\exp[Q_2 \cdot (d_\mathrm{c} - 2x)^2] \cdot \mathit{\Delta}E\}^* = \exp[Q_2^* \cdot (d_\mathrm{c} - 2x)^2] \cdot \mathit{\Delta}E \end{gathered} \qquad \text{(Eq. S10)}$$

which gives the conjugated version of $f$:

$$f^* = -\exp[Q_2^* \cdot (d_\mathrm{c} + 2x)^2] \cdot (\mathit{\Delta}E - 1) + \exp[Q_2^* \cdot (d_\mathrm{c} - 2x)^2] \cdot \mathit{\Delta}E \qquad \text{(Eq. S11)}$$

Substituting the expressions for $f$ and $f^*$ back into the intensity equation, we obtain:

$$\begin{gathered} I_\mathrm{GB} = \left(\frac{w_0}{w}\right)^2 \cdot f \cdot f^* \\ = \left(\frac{w_0}{w}\right)^2 \cdot \exp[(Q_2 + Q_2^*) \cdot (d_\mathrm{c} - 2x)^2] \\ \cdot [\exp(8d_\mathrm{c}Q_2x) \cdot (\mathit{\Delta}E - 1) - \mathit{\Delta}E] \cdot [\exp(8d_\mathrm{c}Q_2^*x) \cdot (\mathit{\Delta}E - 1) - \mathit{\Delta}E] \end{gathered} \quad . \qquad \text{(Eq. S12)}$$

If all the power is concentrated in one of the cores, this equation simplifies to those of the individual Gaussian beams:

$$\begin{aligned} \mathit{\Delta}E = 0 \quad &\rightarrow \quad I_\mathrm{GB} = \left(\frac{w_0}{w}\right)^2 \cdot \exp\left[-\frac{(d_\mathrm{c} + 2x)^2}{2w^2}\right] \\ \mathit{\Delta}E = 1 \quad &\rightarrow \quad I_\mathrm{GB} = \left(\frac{w_0}{w}\right)^2 \cdot \exp\left[-\frac{(d_\mathrm{c} - 2x)^2}{2w^2}\right] \end{aligned} \qquad \text{(Eq. S13)}$$

Of particular interest is the case where the power is equally distributed ($\Delta E = 1/2$), which, after incorporating the above approximate expressions resulting from the assumption $z_\mathrm{R} \ll z$, leads to a simplified version of the intensity:

$$I_\mathrm{GB} = \frac{1}{2}\left(\frac{z_\mathrm{R}}{z}\right)^2 \cdot \exp\left[-\frac{(d_\mathrm{c}{}^2+4x^2)z_\mathrm{R}{}^2}{2w_0{}^2z^2}\right] \cdot \left[\cos\left(\frac{d_\mathrm{c}kx}{z}\right) + \cosh\left(\frac{2d_\mathrm{c}x\, z_\mathrm{R}{}^2}{w_0{}^2z^2}\right)\right] \qquad \text{(Eq. S14)}$$

This equation is an excellent approximation of the situation discussed in this work, as demonstrated by the close overlap between the calculated curve and its numerical counterpart (Fig. S2). From the mathematical form of the equation, several key points can be identified. First, the different oscillating and monotonically varying contributions can be separated, with the width of the distribution mainly defined by the exponential part. The oscillating interference pattern results specifically from the cosine term, which allows an analytic expression for the positions of the local maxima of the intensity distribution. These maxima occur at positions $x_\mathrm{max,m} = m \cdot X_\mathrm{p}$ ($m = 0, \pm 1, \pm 2, \ldots$) with the period of the spatial oscillation:

$$X_\mathrm{p} = \frac{\lambda_0 \cdot z}{d_\mathrm{c} \cdot n_\mathrm{s}} \qquad \text{(Eq. S15)}$$

Using the parameters relevant in the context of this study, $X_\mathrm{p} \approx 5.89\ \mu\mathrm{m}$. The validity of this equation is confirmed by inspecting Fig. S2, where the gray lines indicating $x_\mathrm{max,m}$ coincide with the maxima of the intensity distribution.

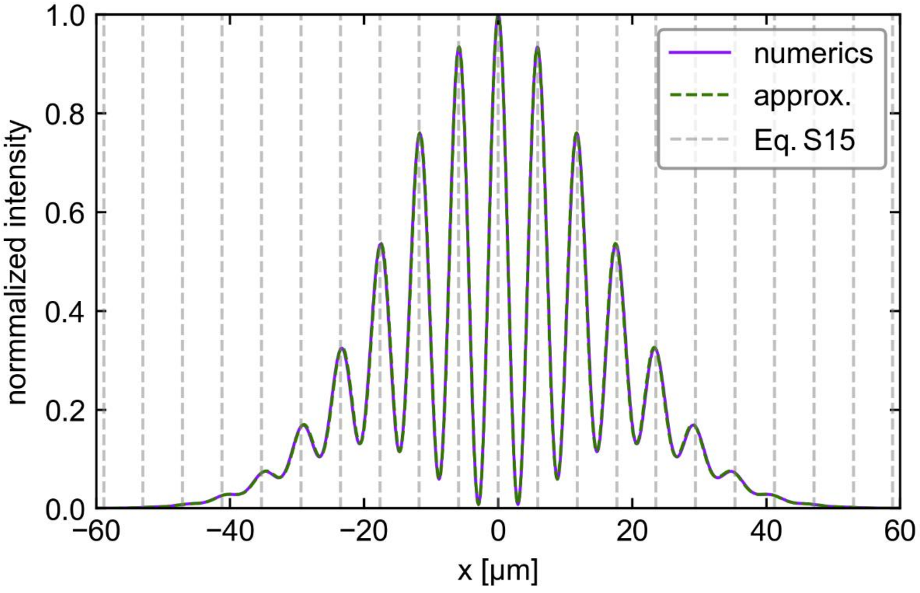


**Figure S2**: **Interference from two Gaussian beams at a defined distance including approximation.** The interference shows such spatial distribution in the lateral plane at a selected distance ($z = 290\ \mu\mathrm{m}$) from the location of the beam waists. The purple solid line refers to the numerical solution (equation S5), the green dashed line to the analytical approximation (equation S14). The calculation parameters are the same as in Fig. S1, including a center-to-center waist distance of $d = 21\ \mu\mathrm{m}$. The vertical dashed gray lines refer to the positions of the maxima calculated by equation S15. Note that the assumed simulation parameters are the same as those used in the main text and in the experiments.

## Supplementary Note 2. Gaussian beam interference

To demonstrate the interference effect between the two Gaussian beams, the intensity distributions perpendicular to the propagation direction were measured at different distances from the DCF surfaces for three different input configurations in an air environment (Fig. S3). As expected, when the input power is fully coupled into a core, a Gaussian intensity distribution is observed at all measured distances (top and bottom rows in figure S3). In the case where the input power is evenly distributed between the two cores, beam

interference becomes noticeable beyond a certain distance (about 80 μm), with the interference pattern becoming more and more pronounced. A very distinct interference pattern is observed at a distance of 187 μm. This distance corresponds to the same beam diameter as in the experiments that include the nanoprinted spacer, which can be explained by the following estimation: Assuming the same beam waist $w(z)$ and $w_0$, and using equation S2 and equation S4, the distances in air and in the spacer medium are related by the expression $z_{\mathrm{air}} = \frac{z_{\mathrm{R}}^{\mathrm{air}}}{z_{\mathrm{R}}^{\mathrm{s}}} z_{\mathrm{s}}$. With a spacer length of $290\ \mu\mathrm{m}$ and the refractive index of IP_DIP2 ($n_{\mathrm{s}}$ at $0.66$ μm = 1.5478), the corresponding distance in air is calculated as $z_{\mathrm{air}} = \frac{290}{1.5478}\ \mu\mathrm{m}\ =\ 187\ \mu\mathrm{m}$.

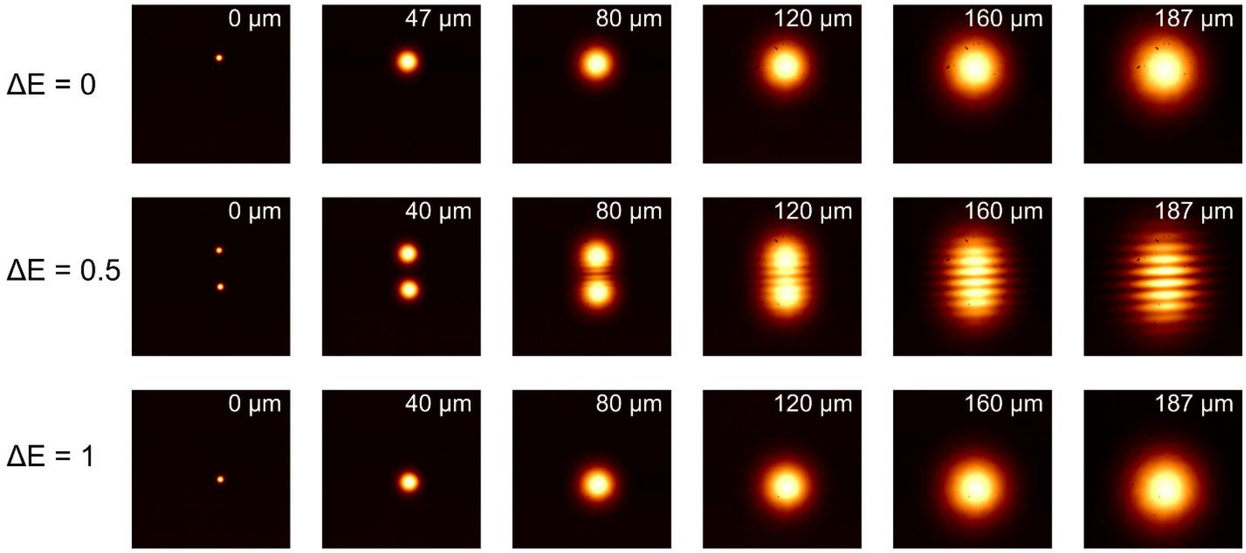


**Figure S3: Measured interference of Gaussian beams.** The intensity distributions were captured at different distances from the DCF surface (indicated by different numbers) for three different input configurations, i.e. relative amplitude difference (top row: $\Delta E\ =\ 0$, middle row: $\Delta E\ =\ 0.5$, bottom row: $\Delta E\ =\ 1$). Each plot is normalized to its maximum value (linear scale. Brightest color: 1, darkest color: 0).

## Supplementary Note 3. Properties of dual core fiber: beam characteristics

Key to the concepts discussed here is the interference of the beams emitted by the modes of the fiber cores at the location of the hologram (HP, $z = z_{\mathrm{f}}$), which requires precise knowledge of the beam properties. To achieve this, the change in beam diameter as a function of distance from the fiber surface in air was measured by taking images at different z-values and fitting the data with the Gaussian beam-width equation $w(z)$ (equation S3, Fig. S4, $\lambda_0 = 660\ \mathrm{nm}$). The diffraction related expansion of the beams is clearly visible, and the highly accurate fit to the data points justifies the approximation of the emitted radiation as Gaussian beams. The agreement between the measured beam waist at the fiber surface ($w_{01} = w_{02} = 1.5\ \mu\mathrm{m}$ at $z = 0\ \mu\mathrm{m}$) confirms that the cores are identical. The resulting divergence angle and numerical aperture were calculated to be $\theta_{\mathrm{div}} = \arctan\left(\frac{\lambda_0}{\pi \cdot w_0}\right) \approx 8°$ and $\mathrm{NA} = \sin(\theta_{\mathrm{div}}) \approx 0.139$.

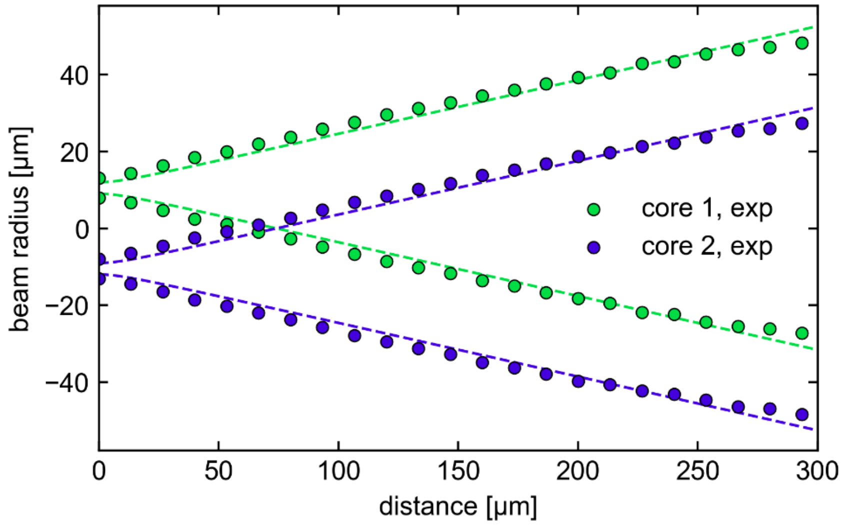


**Figure S4: Measured beam radius as a function of distance from the fiber surface.** The two curves refer to the light emitted by the fundamental modes of each core of the DCF. The points show the experimental data, the dashed lines represent fits to the data points using the Gaussian beam model.

## Supplementary Note 4. Properties of the dual-core fiber: modal attenuation

To determine the single-mode guiding regime of the dual-core fiber used in this study, we measured the spectral distribution of bending-induced losses by simultaneously exciting the fundamental modes in both cores and recording the transmission spectrum while bending the fiber (Fig. S5, fiber length $200$ cm, spectrometer Spectro 320D (Instrument Systems)). The resulting spectrum shows a slight loss peak around $584\ \mathrm{nm}$, which is attributed to the emergence of higher-order modes in the fiber core. For wavelengths longer than $1000\ \mathrm{nm}$, there is a significant increase in bending losses due to insufficient waveguiding (i.e., a very low waveguide parameter). As a result, the practically relevant single-mode regime for this fiber is between $600\ \mathrm{nm}$ and $1000\ \mathrm{nm}$.

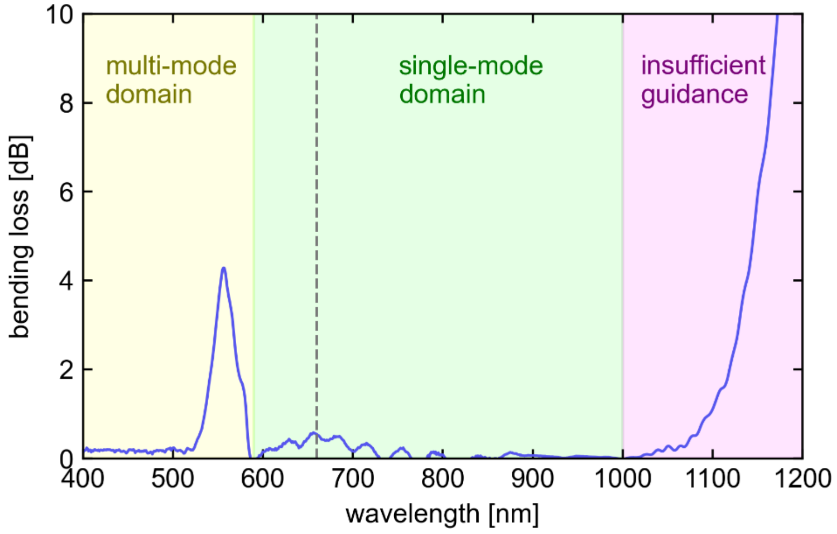


**Figure S5: Spectral distribution of the bending induced loss of the dual-core fiber considered in this work.** The different colors refer to the various guidance regimes. The vertical dashed line indicates the operation wavelength of the experiments used in this work ($\lambda_0 = 660\ \mathrm{nm}$).

## Supplementary Note 5. Conversion between amplitude difference and power difference

The conversion between relative amplitude difference and relative power difference must account for the fact that power is generally related to the square of the modal amplitude, resulting in the following expression:

$$\Delta P = 2\left(\Delta \mathrm{E} - \frac{1}{2}\right)^2 + \frac{1}{2}\begin{cases} +1, & if\ \Delta E > \frac{1}{2} \\ -1, & if\ \Delta E < \frac{1}{2} \end{cases} \qquad \text{(Eq. S16)}$$

## Supplementary Note 6. Details spacer

Structures were printed on the cleaved end face of the DCFs. Note that the spacer section (top diameter $d_\mathrm{s} = 180\ \mu\mathrm{m}$, inset in Fig. 6) was nanoprinted together with the hologram in one run, resulting in a fully monolithic fiber device. Since the spacer itself does not contain any nanostructured elements, relatively coarse printing parameters were chosen to speed up the printing time. To avoid light reflections at the side edges of the spacer, its diameter was chosen to be larger than the fiber diameter ($d_\mathrm{f} < d_\mathrm{s}$). To achieve a significant overlap of the GBs, the distance between the HP and the fiber surface was chosen to be significantly larger than the Rayleigh length ($z_\mathrm{R} \ll z_\mathrm{s}$, $z_\mathrm{s} = 290\ \mu\mathrm{m}$). To improve the mechanical connection between spacer and fiber, the laser exposure required in the printing process was started inside the fiber and a conical section of 50 µm length was printed around the fiber circumference.

## Supplementary Note 7. Optimization of the hologram

*A. Description of the optimization process*

In order to determine configurations that achieve optimum performance, the hologram design balances the following five key criteria to ensure a trade-off between spatial tunability, focus quality, and overall beam shaping efficiency:

1. maximizing spatial modulation,
2. minimizing the full width at half maximum (FWHM) of the focal spot,

3. ensuring identical focal spots for all relative amplitude differences $\Delta E$,
4. suppressing side peak amplitudes, and
5. maximizing spatial separation of the side peaks from the central focal spot.

The optimization process was performed by first considering fixed parameters of the DCF, including the core distance $d_c$ and the waist radius of the Gaussian beams $w_0$. These parameters were first estimated by preliminary simulation and experience, followed by fabrication of a fiber with the defined characteristics. As described in the manuscript, the spots in the focal plane were modeled as small-width Gaussian functions ($w_f = 1\ \mu m$). Optimization was then conducted by calculating the focal pots for seven selected relative amplitude differences $\Delta E$ while varying the spacer length $z_s$ and focal length $z_f$. The optimal configuration was determined using a correlation function that compares the resulting intensity distributions in the focal plane to a Gaussian reference, supplemented by a visual evaluation. Notably, the visual evaluation confirmed that achieving similar FWHM of the focus spots across all seven amplitude differences ensures identical spot formation and a linear dependence of the spatial focus shift on $\Delta E$.

*B. Simulated example configurations*

In order to illustrate the impact of the different parameters on the performance of the focus tuning concept and to highlight the importance for optimising the hologram, two additional configurations with slightly modified parameters were simulated in addition to the experimentally implemented configuration (defined in Tab. S1).

**Table S1: Definition of configurations to illustrate the effect of parameter variations on the intensity distribution in the focal plane.**

| configuration | distance DCF-HP $z_s$ [µm] | distance DCF-IP $z_f$ [µm] | Fig. S6 |
|---|---|---|---|
| used here | 290 | 520 | (a) - (b) |
| A | 150 | 520 | (c) - (d) |
| B | 290 | 250 | (e) - (f) |

The corresponding intensity distributions in the focal plane along the connecting line between the cores (Fig. S6) clearly show that only the optimized structure ensures high-quality focusing over all relative amplitude differences (Fig. S6 (a) - (b)). In contrast, the other two configurations show significant deviations from a Gaussian distribution (Fig. S6 (c) - (d)) or show insufficient spatial tunability ((e) - (f)). Furthermore, for the experimentally implemented configuration, the side lobes remain spatially distant from the central focus, making their influence negligible. However, at shorter focal lengths (Fig. S6 (e) - (f)), the side lobes move closer to the central region, potentially affecting performance.

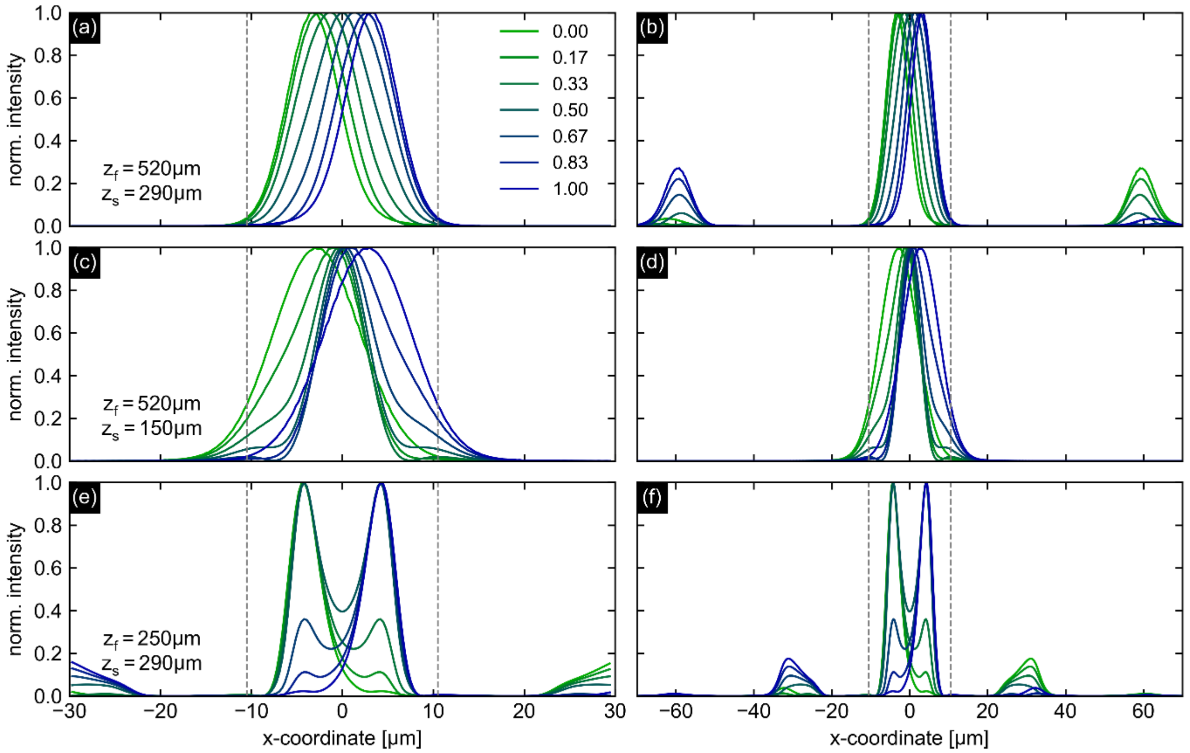


**Fig. S6: Three examples of calculated 1D spatial intensity distributions** along the central line between the two cores in the focal plane for seven different amplitude differences (color-coded as indicated in the legend of (a)), with most parameters taken from the main text. The three examples correspond to different combinations of the DCF-HP and HP-IP distances as given in Tab. S1. Each row shows the results of the same configuration but with different x-axis scales: (**a**)-(**b**) $z_\mathrm{f} = 520\ \mu\mathrm{m}$, $z_\mathrm{s} = 290\ \mu\mathrm{m}$ (experimental configuration), (**c**)-(**d**) $z_\mathrm{f} = 520\ \mu\mathrm{m}$, $z_\mathrm{s} = 150\ \mu\mathrm{m}$, (**e**)-(**f**)

$z_\mathrm{f} = 250\ \mu\mathrm{m}$, $z_\mathrm{s} = 290\ \mu\mathrm{m}$. The vertical gray dashed lines indicate the central positions of the two cores (intercore pitch $d_\mathrm{c} = 21\ \mu\mathrm{m}$).

In summary, optimization of the hologram parameters, taking into account the five criteria described above, is essential to achieve high-quality focus generation and ensure spatial tunability.

## Supplementary Note 8. Discussion of limit of spatial tunability

In this work, achieving optimal performance requires that the hologram design take into account the five key criteria mentioned in the previous Supplementary Note. It should be noted that the influence of various DCF parameters (e.g., core doping concentration, core diameter, and intercore spacing) was not investigated in detail because the limited availability of doped rods with fixed doping levels and geometric parameters did not allow for significant variations in the experiments.

Achieving larger spatial tuning ranges is typically associated with a reduction in focus quality. To illustrate this and to show the effect of core spacing, a parameter sweep was performed for a configuration with a smaller intercore distance $d_\mathrm{c}$ (Fig. S7 (c) - (d)), which shows an increase in spatial tunability at the expense of focus quality. The results are compared with those of the configuration used in this study (Fig. S7 (a) - (b)). While most of the parameters from the main text were kept, the following were changed:

- Distance between source and image plane: $z_\mathrm{f} = 520\ \mu\mathrm{m}$
- Distance from the fiber surface to the hologram: $z_\mathrm{s} = 240\ \mu\mathrm{m}$
- Distance between the cores: $d_\mathrm{c} = 10\ \mu\mathrm{m}$
- Focus position range in the IP: $-7\ \mu\mathrm{m} \leq x_\mathrm{f} \leq 7\ \mu\mathrm{m}$

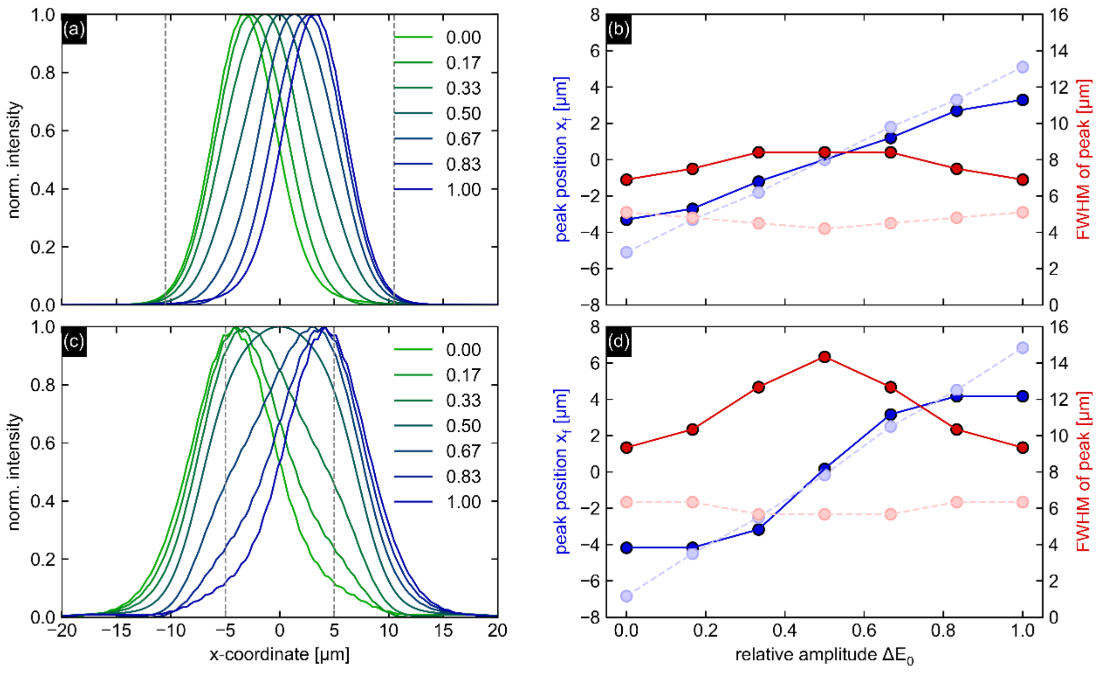


**Fig. S7: Comparison of the focusing properties of the configuration used in this study ((a) - (b), top row) and a configuration that shows a wider tuning range but reduced focusing quality ((c) - (d), bottom row).** The parameters of the latter are defined in the text. (**a, c**) Simulated 1D spatial intensity distributions along the central line between the two cores in the focal plane for seven different amplitude differences (color-coded as indicated in the legend), with most parameters taken from the main text. The vertical gray dashed lines indicate the central positions of the two cores. (**b, d**) Peak position (blue dots, left y-axis) and FWHM (red dots, right y-axis) of each focus as a function of $\Delta E$ (obtained from direct analysis of the data points). The semitransparent blue and red dots refer to the corresponding values obtained from the individual holograms. The solid and dashed lines are included only to connect the dots and serve as a visual guide.

The comparison clearly shows that the alternative configuration allows a larger spatial tuning range (blue dots in Fig. S7(d)). However, the focus shape varies significantly with $\Delta E$ and has a generally larger FWHM (red dots in Fig. S7(d)), indicating a reduction in focus quality. This effect is also evident in the intensity distributions: while the configuration used in this study maintains Gaussian-like profiles across all values of $\Delta E$ (Fig. S7(a)),

the alternative configuration produces partially asymmetric distributions that change in shape (Fig. S7(c)).

In summary, greater spatial tunability can be achieved at the expense of focus quality, thus further simulations, especially considering different DCF designs, are required to fully explore this trade-off.

Note that future research will include more advanced techniques for hologram optimization beyond the Gerchberg-Saxton algorithm used here, which may include novel phase retrieval algorithms with improved properties (e.g., weighted Yang-Gu algorithm (*1*), gradient descent methods (*2*), or deep-learning approaches (*1*)) or inverse design strategies that take into account the peculiarities of 3D nanoprinting (*3*).

## Supplementary Note 9. Spectral dependency

An important effect to consider is the spectral dependence of the tuning focus concept. Apart from the direct wavelength dependence in the relevant equations, the main effect to be considered is the material dispersion of the nanoprinted polymer. To account for this, a well-established Sellmeier equation, which approximates the refractive index of the photoresist used in the nanoprinting process in the visible and near-IR spectral regions (IPDip: $n_{\mathrm{IPDip}} = n_{\mathrm{IPDip}}(\lambda_0)$ (*4*)), is considered in the following.

*A. Effect of wavelength variation on Gaussian beam interference*

A key factor to consider is the influence of wavelength on the interference pattern formed by the two interfering Gaussian beams in the hologram plane. To illustrate this effect, the spatial intensity distribution of the interfering beams along the connecting line of the two fiber cores at the end of the polymer spacer section was calculated for three selected wavelengths (Fig. S8). To emphasize the impact of a different wavelength, the wavelength detuning from the design wavelength ($\lambda_{\mathrm{D}} = 0.66\ \mu\mathrm{m}$) $\Delta\lambda_0 = \lambda - \lambda_{\mathrm{D}}$ is considered in the following.

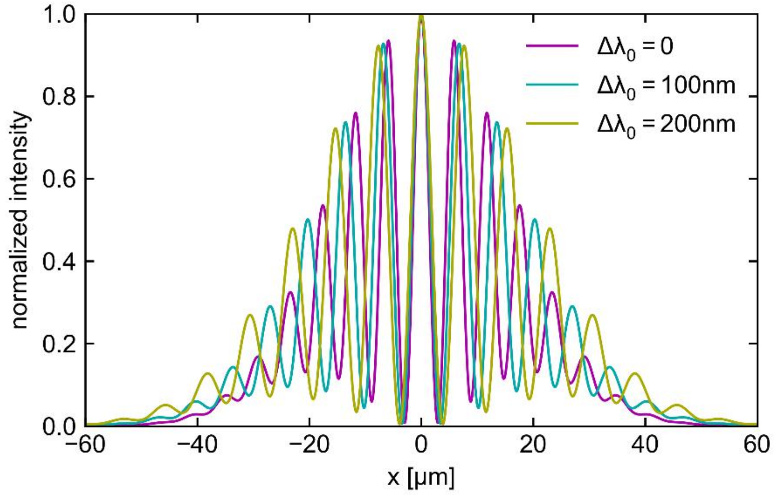


**Fig. S8: Spectral dependence of beam interference.** Simulation of the spatial distribution of the intensity of the two interfering Gaussian beams (parameters taken from the main text) at the end of the polymer spacer section for three selected spectral detunings (magenta: $\Delta\lambda_0 = 0$, cyan: $\Delta\lambda_0 = 100\,\mathrm{nm}$, olive: $\Delta\lambda_0 = 200\,\mathrm{nm}$) in the case of equal power in both cores ($\Delta E = 0.5$).

It is evident that wavelength variations have a significant impact on the spatial intensity distribution. In particular, the more outer regions show substantially different spatial evolutions, suggesting that the intensity profile sensitive focus tuning investigated in this study presumably includes a substantial amount of spectral dependence.

*B. Effect of wavelength variation on intensity distribution in focal plane*

In order to investigate the wavelength dependence of the concept presented here, not only the influence of the wavelength, but also the change of the refractive index of the polymer and thus of the nanoprinted hologram must be considered. This can be accounted for by a correction factor that reflects the fact that a change in wavelength changes the phase distribution due to material dispersion, while the height of the elements remains unchanged. Specifically, during design, the nanoprinted structure was optimized at the design wavelength $\lambda_\mathrm{D} = 0.66\,\mu\mathrm{m}$, and the phase distribution $\Phi_\mathrm{D}$ was converted to a height profile $h(x, y)$ using the following equation:

$$h_\mathrm{D}(x,y) = \phi_\mathrm{D}(x,y) \cdot \frac{\lambda_\mathrm{D}}{n_\mathrm{IPDip}(\lambda_\mathrm{D}) - 1} \quad \text{(Eq. S17)}$$

When the wavelength is changed, the height profile is experimentally preserved, so that the phase profile for a different wavelength $\lambda_0$ changes as follows

$$\phi(x, y, \lambda_0) = \phi_\mathrm{D}(x, y) \frac{\lambda_\mathrm{D}}{\lambda_0} \cdot \frac{n_\mathrm{IPDip}(\lambda_0) - 1}{n_\mathrm{IPDip}(\lambda_\mathrm{D}) - 1} = \phi_\mathrm{D}(x, y) \cdot K \qquad \text{(Eq. S18)}$$

with the correction factor $K$. Following the procedure described in the manuscript, this phase distribution was used to calculate the intensity distributions in the focal plane for seven different amplitude ratios $\Delta E$ using the angular spectrum method in the forward direction.

To illustrate the spectral dependence, Fig. S9 shows the intensity distributions for three different wavelength positive detunings ($\Delta\lambda_0 > 0$). It is evident that the shape of the intensity in the focal plane undergoes significant changes and increasingly deviates from the Airy function, indicating a degradation in focal quality with increasing wavelength. Additionally, the spatial tuning range is reduced with increasing wavelength.

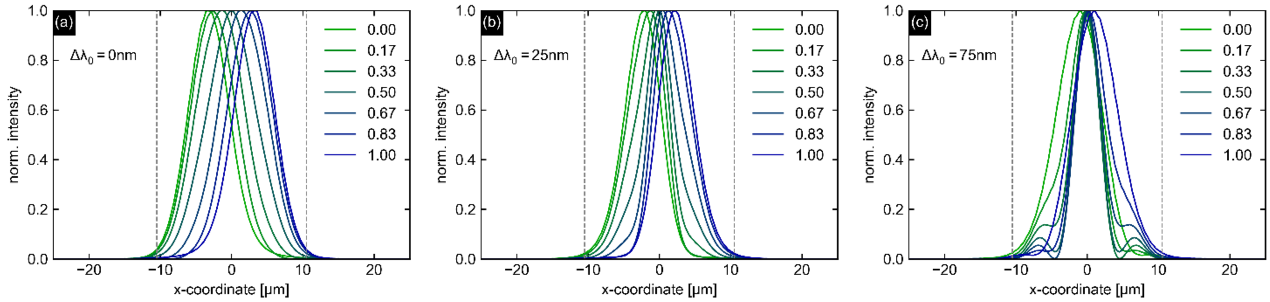


**Fig. S9: Effect of positive spectral detuning on focus tuning.** Three examples of calculated 1D spatial intensity distributions along the central line between the two cores in the focal plane for the seven different amplitude differences (different colors, indicated in the corresponding legend at the top right) (parameters taken from the main text). The three examples refer to three spectral detunings relative to the design wavelength of $\lambda_\mathrm{D} = 0.66\ \mu\mathrm{m}$ towards longer wavelength ((**a**) $\Delta\lambda_0 = 0$ (experimental configuration), (**b**): $\Delta\lambda_0 = 10\ \mathrm{nm}$, (**c**) $\Delta\lambda_0 = 50\ \mathrm{nm}$). The vertical gray dashed lines indicate the central positions of the two cores (intercore pitch $d_\mathrm{c} = 21\ \mu\mathrm{m}$).

The behavior of negative spectral detuning ($\Delta\lambda_0 < 0$), illustrated by example intensity distributions for three different negative detunings in Fig. S10, partially mirrors the trends observed for positive detuning. Specifically, focus quality degrades as the spectral deviation from the design wavelength increases, while the spatial tuning range increases.

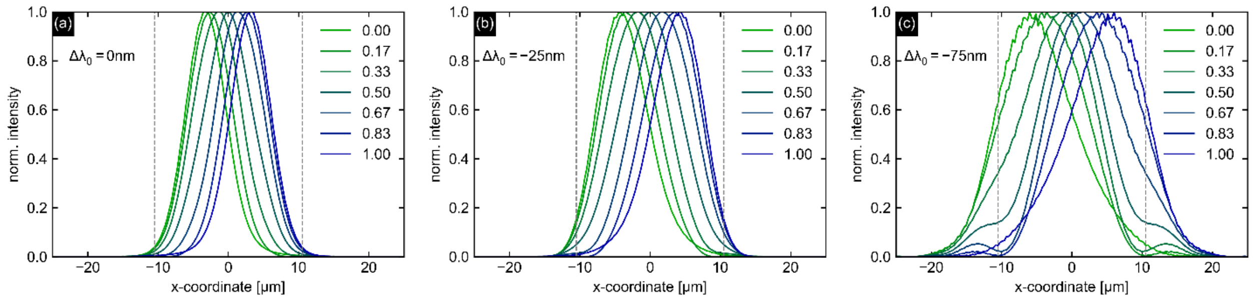


**Fig. S10: Effect of negative spectral detuning on focus tuning.** Calculated examples of 1D spatial intensity distributions along the connecting line between the two cores in the focal plane for the seven different amplitude differences (different colours, indicated in the corresponding legend at the top right). The three examples refer to three spectral detunings relative to the design wavelength of $\lambda_D = 0.66\ \mu m$ towards shorter wavelength ((**a**) $\Delta\lambda_0 = 0$ (experimental configuration), (**b**): $\Delta\lambda_0 = -10\ nm$, (**c**) $\Delta\lambda_0 = -50\ nm$ (gray dashed lines: central positions of the two cores (intercore pitch $d_c = 21\ \mu m$)).

To comprehensively illustrate the wavelength dependence, Fig. S11(a) shows the spatial peak positions of the outermost foci (olive: $\Delta E = 0$, purple: $\Delta E = 1$) along the midline between the two cores in the focal plane as a function of $\Delta\lambda_0$. The results clearly show that the spectral detuning is strongly wavelength dependent, with an increase towards shorter wavelengths. Any deviation from the design wavelength results in a degradation of the focus quality, causing its shape to deviate from an ideal Airy function. This can be seen in Fig. S11(b), which shows the spatial intensity distributions along the line connecting the two cores in the focal plane for different $\Delta\lambda_0$ at a fixed power difference ($\Delta E = 0$). Only the curve that is related to the design wavelength ($\Delta\lambda_0 = 0$, thick red line) resembles a symmetrical Airy function profile.

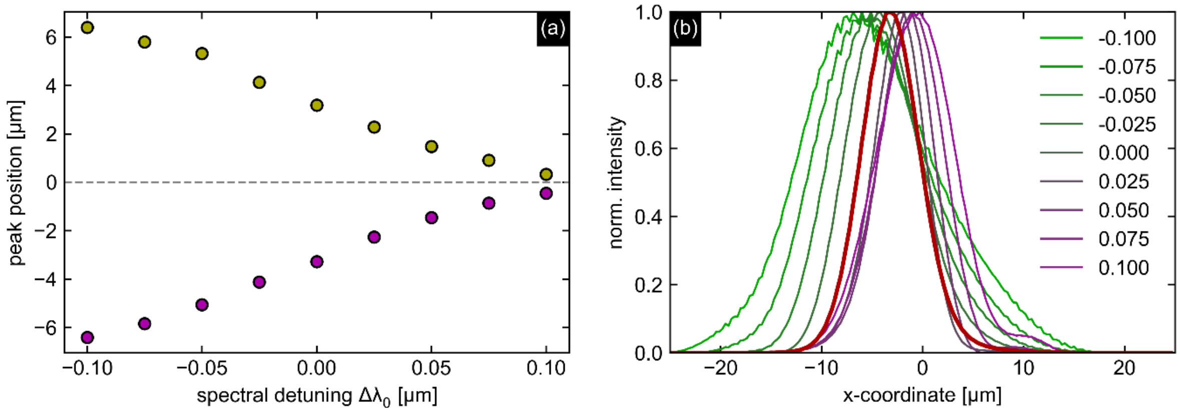


**Fig. S11: Presentation of the key features of the spectral analysis.** (**a**) Spatial positions of the outermost foci (olive: $\Delta E = 0$, purple: $\Delta E = 1$) along the central line between the two cores in the focal plane as a function of spectral detuning from the design wavelength $\lambda_{\mathrm{D}}$ (parameters taken from the main text). The horizontal grey dashed lines indicate the central position ($x = 0$). (**b**) Spatial intensity distributions along the line connecting the two cores in the focal plane for $\Delta E = 0$, shown for nine different spectral detunings (indicated in the legend). The curve corresponding to the design wavelength ($\Delta\lambda_0 = 0$) is highlighted with a thicker red line for clarity.

Note that the primary focus of this work is the spatial tuning of a high-quality focus. Regarding the possible bandwidth, the above discussion reveals that the desired performance is maintained over a total bandwidth of $100\ \mathrm{nm}$.

## Supplementary Note 10. Approximation of focus by Airy function

The Airy function is widely used in optics to describe the diffraction pattern of a circular aperture and is a well-established approximation for an ideal focus in systems governed by diffraction. Its use in this study ensures an accurate representation of the simulated focal spot and allows a meaningful comparison between experimental and theoretical results. In the following, we will review the main steps of the deviation of the Airy function in the context of light focusing.

*Step 1: Fraunhofer diffraction approximation*

The starting point is the Fraunhofer diffraction approximation, which is valid in the far field (or in the focal plane of a lens). In this approximation, the electric field at an observation point is given by the Fourier transform of the aperture (or pupil) function. Mathematically, the electric field is expressed as

$$E(x,y) \propto \iint A(x',y') \cdot \exp\left[-i \cdot \frac{2\pi}{\lambda_0 z} \cdot (x \cdot x' + y \cdot y')\right] dx'dy' \qquad \text{(Eq. S19)}$$

where $A(x',y')$ represents the amplitude and phase distribution across the aperture.

*Step 2: Conversion to polar coordinates:*

Since the aperture is circular, it is convenient to change from Cartesian to polar coordinates. The aperture coordinates $(x',y')$ are expressed as $(r' \cdot \cos\theta, r' \cdot \sin\theta)$, and similarly, the observation coordinates $(x,y)$ become $(r \cdot \cos\varphi, r \cdot \sin\varphi)$. This conversion simplifies the integral, especially the angular part, which contains an exponential term with a cosine function that can be simplified using the identity

$$\int_0^{2\pi} \exp(-ikr' \cdot \cos\theta)\, d\theta = 2\pi \cdot J_0(kr') \qquad \text{(Eq. S20)}$$

with zeroth-order Bessel function $J_0$.

*Step 3: Radial integration*

The next step involves integration over the radial coordinate $r'$ from 0 to the aperture radius $R$, which has the form:

$$E(x,y) \propto \int_0^R \mathrm{r}' \cdot J_0\left(\frac{2\pi r'}{\lambda_0 z}\right) dr' \qquad \text{(Eq. S21)}$$

Evaluating this integral leads to an expression for electric field distribution that includes the first-order Bessel function $J_1$:

$$E(r) \propto \frac{R\lambda_0 z}{2\pi r} J_1\left(\frac{2\pi Rr}{\lambda_0 z}\right) \propto \frac{1}{\frac{2\pi Rr}{\lambda_0 z}} J_1\left(\frac{2\pi Rr}{\lambda_0 z}\right) = \mathrm{jinc}\left(\frac{2\pi Rr}{\lambda_0 z}\right) \qquad \text{(Eq. S22)}$$

showing that the intensity is proportional to the square of the jinc-function.

## Supplementary Note 11. Temperature influence

To demonstrate the effect of temperature on the focus tuning concept, the relevant temperature range is first defined and then the different types of effects are systematically discussed step-by-step.

*A. Definition of key parameters*

As the silica-based DCF remains mechanically stable up to very high temperatures ($> 1500\ °\mathrm{C}$), the upper temperature limit is primarily determined by the onset of structural deformation in the nanoprinted polymer. Based on previous studies and literature, this occurs around $T_{\mathrm{up}} \approx 100\ °\mathrm{C}$. The lower temperature limit is more application dependent and less strictly defined. Without loss of generality, we choose $T_{\mathrm{low}} = 20\ °\mathrm{C}$ as this value is commonly used as the reference temperature $T_0$ for defining relevant material coefficients.

To determine the effect of temperature on device performance, it is essential to consider the thermo-optical properties of the materials involved, characterised by the thermo-optical coefficient (TOC), which quantifies the change in refractive index with temperature as follows:

$$n(\lambda_0, T) = n(\lambda_0, T_0) + \beta \cdot (T - T_0) \quad \text{(Eq. S23)}$$

with the TOC $\beta$, the temperature $T$ and the vacuum wavelength $\lambda_0$. In this study the effect of temperature must be considered for two key materials:

- silica (Tab. 1 of Ref. (*5*)): $\beta_{\mathrm{silica}} = 8.38 \cdot 10^{-6}\ K^{-1}$
- polymer (IP-DIP, Ref. (*6*)): $\beta_{\mathrm{IPDip}} = -3 \cdot 10^{-4}\ K^{-1}$

For the nanoprinted structure, we also consider a possible temperature-dependent change in the length of the elements, characterised by the coefficient of thermal expansion (CTE) along one dimension, given by

$$L(T) = L_0 \cdot [1 + \alpha(T - T_0)] \quad \text{(Eq. S24)}$$

Where $L(T)$ is the length at temperature $T$, $L_0$ is the reference length at $T_0$, and $\alpha$ is the CTE. For nanoprinted polymers this coefficient is given by $\alpha_{\mathrm{IPDip}} = 2.9 \cdot 10^{-4} K^{-1}$ (*6*).

*B. Impact on modes inside the DCF*

To uncover the effect of temperature on mode formation within the DCF, the waveguide parameter $V$, a key indicator of the waveguide system, was calculated for both temperatures considered. The results show that due to the minimal change in the refractive index of the silica, the variation in the parameter from $T_{\mathrm{low}}$ to $T_{\mathrm{up}}$ is significantly less than $0.1\%$, indicating that the modes within the DCF remain virtually unaffected. This is crucial as it confirms that the mode field diameter of the Gaussian beams remains unchanged, meaning that $w_0 = 1.5\ \mu\mathrm{m}$ can be used in both cases.

*C. Impact on Gaussian beams*

Another important factor to consider is the variation in the interference patterns of the two Gaussian beams overlapping in the hologram area. In contrast to silica, the polymer has a moderately high TOC, which leads to a practically relevant change in the refractive index. However, the corresponding change in the Rayleigh length of the Gaussian beams remains negligible for the application targeted in this work (Tab. S2).

**Table S2: Key parameters for the Gaussian beam propagation at the two temperatures considered.**

| parameter | value at $T_{\mathrm{low}}$ | value at $T_{\mathrm{high}}$ |
|---|---|---|
| refractive index of medium | 1.5478 | 1.5238 |
| Rayleigh length [μm] | 16.58 | 16.32 |

To understand the effect of temperature on beam interference, the spatial distribution of the light intensity of the interfering Gaussian beams along the connecting line of the two fiber cores at the end of the polymer spacer section was calculated for both temperatures (Fig. S12). The results show that the curves overlap almost completely, indicating

that any temperature effect on the operating principle itself, i.e. intensity profile based control, can be safely neglected.

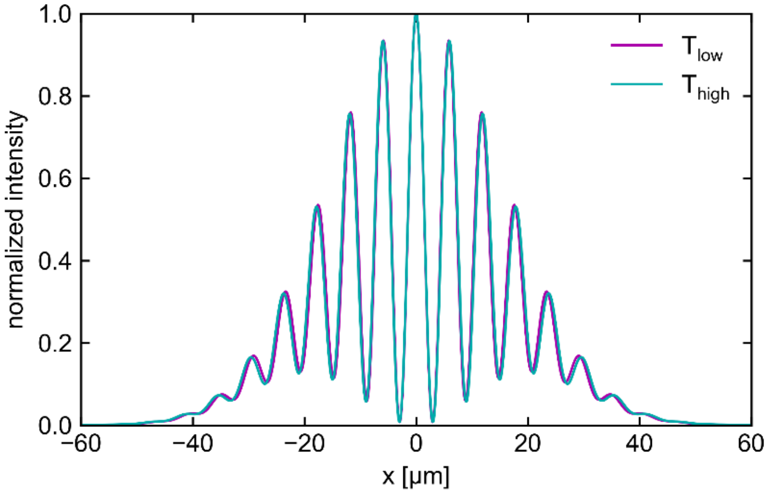


**Fig. S12: Temperature dependence of beam interference.** Simulation of the spatial distribution of the intensity of two interfering Gaussian beams (parameters taken from the main text) at the end of the polymer spacer section for the two discussed temperatures (purple: lower temperature limit $T_{\mathrm{low}}$, cyan: upper temperature limit $T_{\mathrm{high}}$).

*D1: Temperature influence: thermo-optic effect*

A temperature induced change in the refractive index of the nanoprinted elements requires compensation by an adjustment in the elements' height. As shown in the manuscript, the element height is generally given by

$$h = \phi \frac{\lambda_0}{n_{\mathrm{IPDip}} - 1} \qquad \text{(Eq. S25)}$$

with the refractive index of the polymer $n_{\mathrm{IPDip}}$ and the phase distribution of the hologram $\phi$. At this stage it is important to note that $\phi$ itself does not depend on temperature, which can be illustrated by a kinoform profile for a lens operating in air, given by

$$\phi = -n_{\mathrm{air}} \cdot k_0 \left( \sqrt{r^2 + f^2} - f \right) \qquad \text{(Eq. S26)}$$

with the refractive index of air $n_{\text{air}}$, the vacuum wave vector $k_0$, the radial coordinate $r$, and the focal length $f$. Since the refractive index of air is effectively constant over the temperature range considered ($n_{\text{air}} \approx 1$), the phase remains unchanged with temperature. As a result, the maximum height compensation required due to the thermo-optical effect can be expressed as

$$\delta h_{\max}^{\text{TOC}} = \frac{h(T_{\text{high}})}{h(T_0)} = \frac{n_{\text{IPDip}}(\lambda_0 \cdot T_0) - 1}{n_{\text{IPDip}}(\lambda_0 \cdot T_{\text{high}}) - 1} \approx -4.6\% \qquad \text{(Eq. S27)}$$

For the purposes of this study, assuming an element height of $1.2\ \mu\text{m}$ at $T_0$ (corresponding to a $2\pi$ phase shift), a height reduction of *55* nm is required. This value is small compared to the vertical pixel resolution of the nanoprinted voxel along the vertical direction and can safely be neglected.

*D2: Temperature influence: thermal expansion*

A similar type of investigation can be carried out to determine the maximum height compensation required to account for changes in element length due to thermal expansion, leading to the definition of the relative compensation height:

$$\delta h_{\max}^{\text{TEC}} = \frac{h(T_{\text{high}})}{h(T_0)} \approx -2.32\% \qquad \text{(Eq. S28)}$$

The resulting value is even smaller than that obtained from the thermo-optical analysis, indicating that this effect is practically negligible.

Overall, the analysis confirms that temperature effects can be safely neglected within the relevant range. It is important to note that the upper temperature limit considered here ($T_{\text{high}} = 100\ ^\circ\text{C}$) is significantly higher than typically required in many practical applications. As most practical applications have much lower operating temperatures, the actual impact of temperature variation is even less than estimated.

It should be noted that the incoupling of light into the fiber cores on the input side may require readjustment if the temperature varies significantly, especially for long-term measurements. Based on our extensive experience in coupling light into fibers with substantially more complex cross-sections (e.g., single-element anti-resonance fibers (*7*) and input configurations (e.g., higher-order modes in liquid core fibers (*8*)), the current

setup has been optimized to ensure stable coupling conditions over several days, enabling highly reproducible measurements. Future improvements in coupling stability may be achieved by integrating the system into fiber circuitry.

## Supplementary Note 12. Summary of the symbols used in this work and their explanation

**Table S3: Overview of the parameters and their values used in the experiments and simulations.**

| parameter | symbol | unit | value |
|---|---|---|---|
| operation vacuum wavelength | $\lambda_0$ | µm | 0.66 |
| distance between hologram plane and image plane | $z_\mathrm{f}$ | µm | 520 |
| spatial resolution of the nanoprinter | $\Delta x, \Delta y$ | µm | 0.3 |
| refractive index of spacer at $\lambda_0$ | $n_\mathrm{s}$ | 1 | 1.547794 |
| refractive index of the polymer at $\lambda_0$ | $n_\mathrm{p}$ | 1 | 1.547794 |
| beam waist of the Gaussian beam | $w_0$ | µm | 1.5 |
| thickness spacer (i.e., distance fiber surface-hologram) | $z_s$ | µm | 290 |
| distance between centres of fiber cores | $d_\mathrm{c}$ | µm | 21 |
| diameter of spacer | $d_\mathrm{s}$ | µm | > 120 |
| outer diameter of the dual-core fiber | $d_\mathrm{f}$ | µm | 160 |
| amplitude Gaussian beam 1 | $E_1$ | 1 | 0. . . 1 |
| amplitude Gaussian beam 2 | $E_2$ | 1 | 0. . . 1 |
| relative amplitude difference | $\Delta E$ | 1 | 0. . . 1 |
| refractive index of cladding at $\lambda_0$ | $n_\mathrm{cl}$ | 1 | 1.4563 |
| refractive index of core at $\lambda_0$ | $n_\mathrm{co}$ | 1 | 1.4623 |
| numerical aperture of cores | NA | 1 | 0.14 |
| position of focus in the IP | $r_\mathrm{f} = (x_\mathrm{f}, y_\mathrm{f})$ | (µm, µm) | (-5. . . 5, 0) |
| No. of foci in IP considered | $N_\mathrm{f}$ | 1 | 7 |
| radius of the considered GBs in the IP | $w_\mathrm{f}$ | µm | 1 |
| phase distribution of individual hologram | $\phi_i$ | 1 | - |
| phase distribution of combined hologram | $\phi_\mathrm{hol}$ | 1 | - |
| FWHM of focus (fitted) | FWHM | µm | - |
| core diameter | $d_\mathrm{co}$ | µm | 3.4 |

## References of Supplementary Information


1. Mouthaan, R. *et al*. Generating High-Fidelity Structured Light Fields Through an Ultrathin Multimode Fiber Using Phase Retrieval, *Advanced Optical Materials* **13**, 2401985 (2025).

2. Liu, S. & Takaki, Y. Gradient descent based algorithm of generating phase-only holograms of 3D images, *Opt. Express* **30**, 17416-17436 (2022).

3. Augenstein, Y. & Rockstuhl, C. Inverse Design of Nanophotonic Devices with Structural Integrity, *ACS Photonics* **7** (8), 2190-2196 (2020).

4. Jang, B. *et al.* Light guidance in photonic band gap guiding dual-ring light cages implemented by direct laser writing, *Opt. Lett.* **44**, 4016-4019 (2019).

5. Rego G. Temperature Dependence of the Thermo-Optic Coefficient of $SiO_2$ Glass. *Sensors* **23**(13), 6023 (2023).

6. Smith, J.W. *et al*. Three-dimensional Fabry–Pérot cavities sculpted on fiber tips using a multiphoton polymerization process, *Micromech. Microeng.* **30**, 125007 (2020).

7. Nissen, M. *et al*. Nanoparticle Tracking in Single-Antiresonant-Element Fiber for High-Precision Size Distribution Analysis of Mono- and Polydisperse Samples, *Small* **18**, 2202024 (2022)

8. Hofmann, J., Scheibinger, R. & Schmidt, M. A. Characterizing temporal stability of supercontinuum generation in higher-order modes supported by liquid-core fibers, *Sci. Rep.* **14**, 23947 (2024).
